# Development and evaluation of a free e-learning program on dementia risk reduction for the general public: A pre-post study

**DOI:** 10.1177/13872877241309112

**Published:** 2025-01-10

**Authors:** Stephanie Van Asbroeck, Sophie CPM Wimmers, Martin PJ van Boxtel, Rob BM Groot Zwaaftink, Vera Otten, Dinant Bekkenkamp, Sebastian Köhler, Kay Deckers

**Affiliations:** 1 Alzheimer Centrum Limburg, Mental Health and Neuroscience Research Institute (MHeNs), Department of Psychiatry and Neuropsychology, Maastricht University, Maastricht, Netherlands; 2 Alzheimer Nederland, Amersfoort, Netherlands

**Keywords:** Alzheimer's disease, dementia, education, healthy lifestyle, health behavior, learning, primary prevention, risk factors, telemedicine

## Abstract

**Background:**

There is consistent evidence for the contribution of modifiable risk factors to dementia risk, offering opportunities for primary prevention. Yet, most individuals are unaware of these opportunities.

**Objective:**

To investigate whether online education about dementia risk reduction may be a low-level means to increase knowledge and support self-management of modifiable dementia risk factors.

**Methods:**

A pre-post study was conducted with Dutch community-dwelling individuals who registered for a free e-learning course called “Keep your brain healthy”. The e-learning covers seven themes delivered week-by-week covering cognitive and physical activity, diet, and cardiovascular health, amongst others. Participants completed an online survey before starting the e-learning, immediately afterwards, and three months later. The survey covered user experience, knowledge on dementia risk reduction, motivation for, and engagement in, health behaviors.

**Results:**

Of the 477 participants (70.9% women, mean age = 63 years), 339 (71.1%) completed the survey immediately after the e-learning, and 241 (50.5%) completed the three-month follow-up survey. User experiences were positive with weekly themes receiving average ratings between 7.9–8.1 out of 10. Improvements over time were seen in knowledge of dementia risk reduction, Mediterranean diet adherence, social contact satisfaction, and motivation for physical activity. Cognitive activity levels and alcohol consumption improved over time in women. Moreover, improvements in knowledge and Mediterranean diet adherence remained present three months after course completion.

**Conclusions:**

This e-learning program was positively perceived, increased knowledge of dementia risk reduction, and promoted engagement in brain-healthy lifestyles. The program can easily be implemented as a stand-alone tool or as part of larger dementia risk reduction initiatives.

## Introduction

The number of people living with Alzheimer's disease and related dementias worldwide is expected to increase by more than 2.5-fold by 2050, making the syndrome a public health priority.^1^ It has recently been estimated that potentially 45% of all dementia cases worldwide can be attributed to 14 modifiable risk factors, such as depression, education level and hearing loss. The management of these risk factors is complex, and to bring about significant reduction of these risk factors, (inter)national policy change, health programs and individually tailored interventions are required.^2^ Therefore, reducing dementia risk by promoting a “brain-healthy” lifestyle offers considerable opportunities.^3^ However, many people are unaware of the potential for dementia risk reduction. Especially cardiovascular risk factors like hypertension, high cholesterol and obesity are very poorly recognized.^4–6^ Yet, most individuals (>70%) indicate they would want to learn more about the topic.^7–9^ Raising awareness is an important first step towards conscious behavior change and may empower individuals.^8–10^ The knowledge that a set of lifestyle behaviors is also beneficial for your current cognitive- and brain health is new for most individuals and may also provide additional motivation for making actual behavior changes.^8^

E-learning is a low-level means to increase knowledge and awareness, can potentially have a large reach, and may be easily implemented as a stand-alone tool or as part of current or future dementia risk reduction initiatives if proven effective and well-perceived.^11–13^ Online education can positively impact knowledge about health promotion for various non-communicable (and communicable) diseases, but has also been shown to positively affect attitudes, intentions, health behaviors, and quality of life.^14–16^ However, evidence on the efficacy of online education on behavior change and health outcomes in the general public is limited.^16^ There are many studies in which an eHealth tool is evaluated by self-reported perception of change in knowledge, motivation, or behavior, surveyed only after use. These measures are often biased and not validated to give information on actual health behavior (change).^17,18^

Studies have shown that individuals would appreciate information on dementia risk reduction via eHealth tools.^4–6,19^ A few eHealth tools for raising awareness (and supporting behavior change) towards dementia risk reduction have been developed and are currently available for use. For example, Alzheimer Nederland (the Dutch Alzheimer's society) introduced in 2021 e-learning called ‘Keep your brain healthy’ consisting of weekly emails with facts and tips on a brain-healthy lifestyle. Another example is a comprehensive massive open online course (MOOC) on dementia prevention developed by the Wicking Dementia Research and Education Centre at the University of Tasmania.^20^ Smartphone apps on dementia risk reduction have also been developed (e.g., ‘MyBraincoach’), that have been shown to benefit users’ knowledge on dementia risk reduction.^21^ Yet, these existing tools did not include appealing infographics, general information on brain functioning/cognitive aging and lifestyle coach-guided advice on how to make sustainable lifestyle changes.

To answer the demand for evidence-based information about dementia risk reduction, we refined and extended a free, comprehensive, online e–learning tool about dementia risk reduction for the Dutch general adult population. The aim was to thoroughly evaluate this low-level intervention by assessing potential changes in participants’ knowledge, health behaviors and motivation for health behaviors over time, by administering predominantly validated questionnaires at baseline and follow-up. Further, user experiences and usage of the e-learning materials were also examined.

## Methods

### Design and participants

**Figure 1.**
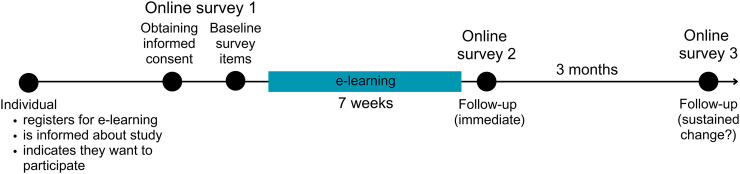
Study flow chart.

This is a single-group pre-post study with individuals from the general population who registered for a Dutch e-learning about dementia risk reduction. Facebook advertisements have also been used (including during the recruitment period) to draw individuals to the e-learning. Recruitment ran from November 15, 2023, until December 27, 2023. Data collection was finalized on June 5, 2024. After providing (online) informed consent, online surveys were administered before starting the e-learning, immediately afterwards (i.e., seven weeks after starting the e-learning), and again three months later (Figure 1). The primary outcome was change in knowledge of dementia risk/protective factors before versus after completion of the e-learning. Secondary outcomes consisted of a description of the user experience and satisfaction with the e-learning, as well as change in health behaviors and motivation for health behaviors before versus after. Based on a priori power analysis (effect size = 2, mean knowledge score_before _= 35, standard deviation = 9, two-sided testing, alpha = 0.05, power = 0.90, drop-out rate of 50% by the last survey based on previous studies with a comparable design), a sample of 430 individuals was considered sufficient to detect a two-point difference in terms of knowledge of dementia risk/protective factors over time. This study received ethical approval from the Ethics Review Committee Faculty of Health, Medicine and Life Sciences (FHML-REC) of Maastricht University (reference number: FHML-REC/2023/103). All participants gave informed consent.

### ‘Keep your brain healthy’ e-learning

The e-learning evaluated in this study was collaboratively developed from a pre-existing e-learning of Alzheimer Nederland and builds on the latest insights from the dementia risk reduction field provided by researchers from the Alzheimer Centum Limburg of Maastricht University. The pre-existing e-learning already had a large reach, with about 35,000 registrations per year. After a needs assessment (online survey filled in by 172 individuals), in which previous users of the pre-existing e-learning were surveyed, the current updated and extended e-learning was developed. More details about, and results of, the needs assessment can be found in Supplemental Material 1.

**Figure 2.**
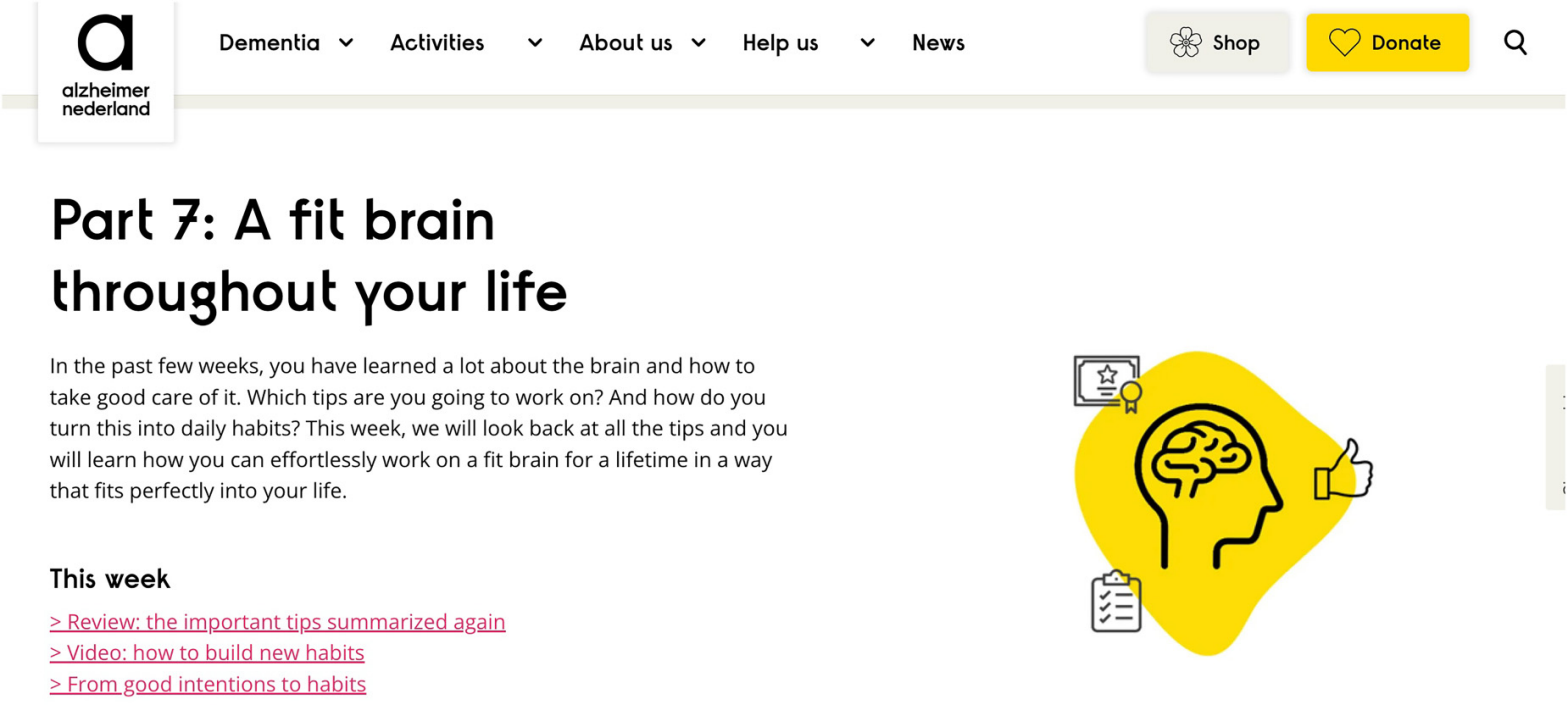
Screenshot of the webpage of part 7 of the e-learning about making sustainable lifestyle changes (translated to English).

The e-learning consists of seven parts that are delivered via weekly e-mails. The seven parts cover the following topics: (1) how the brain works and cognitive changes during aging, (2) cognitive and social activity, (3) healthy diet and alcohol consumption, (4) physical activity, (5) mental wellbeing, including sleep, (6) cardiovascular health, and (7) lifestyle coach-guided advice on how to make sustainable lifestyle changes (Figure 2).

**Figure 3.**
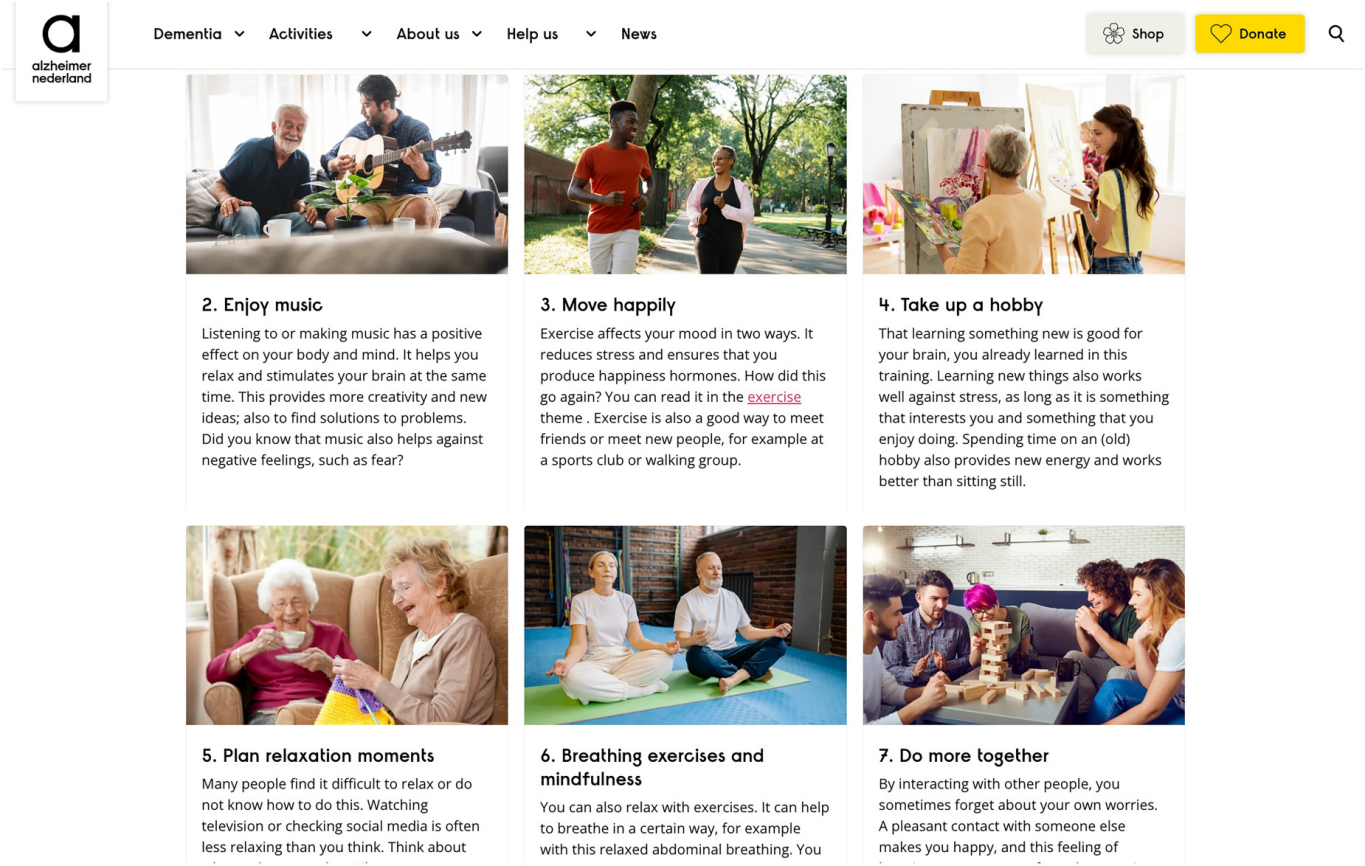
Screenshot of a webpage of the e-learning (translated to English).

**Figure 4.**
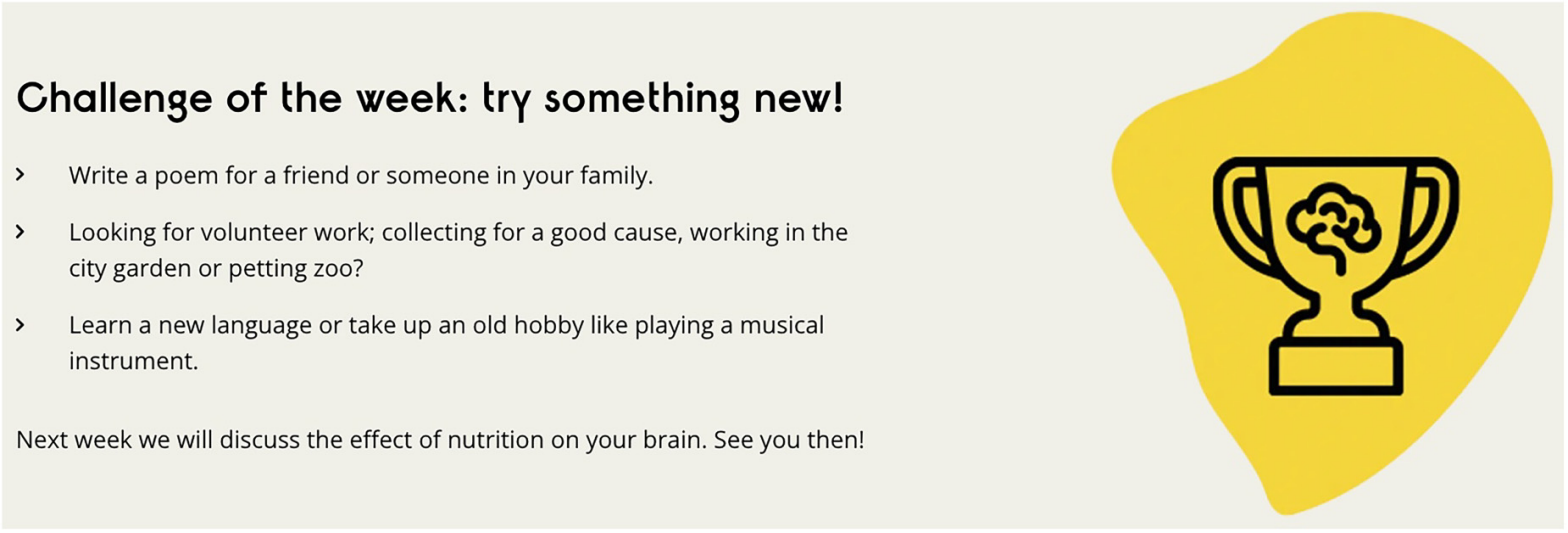
An example of a weekly challenge (translated to English).

The weekly e-mail guides users first to a short knowledge quiz, and thereafter to a webpage containing further information, including a video, and practical tips (Figure 3). The quiz was the central element and designed to cover all essential learning goals. Users are also free to skip the quiz and go directly to the webpage. The e-mail further contains a selection of three weekly challenges (ranging in difficulty from light to hard) as an immediate call to action (Figure 4).

More e-learning content can be found in Supplemental Material 2. The minimum duration of the e-learning is seven weeks, but the user is free to decide whether and how fast they go through the materials. The e-learning is completely online. There is no personal contact between the “learners” or “teachers”, neither are there tests or exams.

### Measures

This study employed online surveys to evaluate user experience (i.e., ease of use, attractiveness and enjoyability, educational and motivating quality, meeting expectations), engagement, and satisfaction immediately after the seven-week e-learning period. Changes over time in terms of knowledge of dementia risk reduction, motivation for health behaviors, and actual health behaviors themselves, were examined. Demographical information was also collected. The surveys were administered via Qualtrics XM. The complete translated surveys can be found in Supplemental Material 3.

### Demographics and other characteristics

Age, gender, educational level, and personal familiarity with dementia (i.e., yes/no answer to the question “Have you ever personally known anyone with dementia or have it yourself?”) were surveyed. Educational attainment was further categorized into three levels. Primary school or low vocational education were categorized into low level of education. Intermediate secondary education, intermediate vocational, or higher secondary education were categorized into medium level of education. Higher vocational education or a university degree were categorized as high level of education.

### Knowledge of dementia risk reduction

General awareness of the potential for dementia risk reduction was surveyed as well as knowledge of 15 well-established modifiable dementia risk and protective factors (i.e., high cognitive activity, low-to-moderate alcohol consumption, healthy diet, physical inactivity, smoking, low social participation, hypertension, dyslipidemia, diabetes, obesity, hearing impairment, sleep disturbances, chronic kidney disease, coronary heart disease, and depression).^4,6,19,22,23^ Individuals disagreeing or disagreeing strongly with the statement ‘There is nothing anyone can do to reduce their risks of getting dementia’ (on a 5-point Likert scale ranging from ‘strongly disagree’ to ‘strongly agree’) were considered to be aware of dementia risk reduction. Knowledge of individual risk and protective factors was also assessed via statements (e.g., ‘High blood pressure increases your chances of getting dementia’) to which participants could respond on a 5-point Likert scale. All statements on risk and protective factors were written in correct form, thus agreement with the statement would indicate knowledge of the risk/protective factor. Overall knowledge of dementia risk reduction was operationalized as the sum score across all 16 statements (individual risk/protective factors and general awareness of dementia risk reduction). ‘Strong agreement’ with a correct statement resulted in four points being added to the participant's score, ‘agreement’ resulted in three points being added, and so on. Strong disagreement resulted in zero points. For the (incorrect) statement on general dementia risk reduction awareness, the scoring was inverse to that described above.

### Motivation for health behaviors

The surveys further included the Motivation To Stop Scale^24^ on motivation for smoking cessation, the Reduce Alcohol Consumption Scale^25^ on motivation for reducing alcohol consumption and custom items on motivation for engaging in physical activity and adhering to a healthy diet.

### Health behaviors

Items were included on alcohol consumption (questions from the Alcohol Use Disorders Identification Test (AUDIT)),^26^ smoking (questions from the Global Adult Tobacco Survey (GATS)),^27^ and weight and height (to calculate body mass index (BMI)). Nine items based on the Cognitive Activity Leisure Scale^28^ were used to measure cognitive activity. These items were scored by assigning a point to each answer (ranging from ‘Never’ = 0 to ‘Daily’ = 5) and summing these values over all activities. The European Prospective Investigation into Cancer and Nutrition (EPIC) Physical Activity Questionnaire^29,30^ was used to assess physical activity, and the Cambridge Physical Activity Index was used to categorize individuals into four levels of physical activity (inactive, moderately inactive, moderately active, active).^30^ Single custom-made items were used to assess adherence to the Mediterranean diet, satisfaction with social contacts, sleep quality, and mood (by rating on a visual analogue scale (VAS) ranging between 0–10). Participants were asked at baseline about a having received a diagnosis of dyslipidemia, cardiovascular disease, hypertension, diabetes, and chronic kidney disease. At the immediate and 3-month follow-up, they were asked whether they took actions to improve their blood pressure, cholesterol levels, or glycaemia.

### Modifiable dementia risk

The LIfestyle for BRAin health (LIBRA) score was calculated. This is a comprehensive, composite risk score that summarizes the presence or absence of twelve modifiable risk/protective factors for dementia into one numeric value.^31,32^ It has been extensively validated to predict incident dementia, cognitive decline and has been associated with biomarkers of brain damage.^33–38^ Moreover, LIBRA has been used as an outcome measure in multiple randomized intervention trials.^21,39^ For each risk/protective factor that is present within an individual a predefined value/weight gets added to the score. Here, someone was considered cognitively active with a total cognitive activity score >30. Individuals who drank no more than seven standard units of alcohol per week, were considered low-to-moderate alcohol consumers.^40^ Individuals who self-rated their adherence to a Mediterranean diet as high (≥8 out of 10) were considered to adhere to the Mediterranean diet. Obesity was defined as a BMI ≥ 30 kg/m^2^.^41^ Lastly, agreement of less than 6 out of 10 to the statement about mood (‘Generally, I feel cheerful and find life worthwhile’, with 0 = ‘completely disagree’ to 10 = ‘completely agree’) was considered indicative of potential depression. Further details on the calculation of LIBRA can be found elsewhere.^38^ A higher LIBRA score is indicative of a worse combination or risk/protective factors.

### Engagement with the e-learning

Google Analytics, Hotjar, Spotler, Riddle, and Youtube were used to monitor group level engagement data of regular non-participating users of the e-learning, and our study participants. Where possible, non-participating user data was limited to the same period when study participants were receiving the e-learning (16 November 2023 to 16 February 2024). This data included the number of opened e-mails, number of clicks to the quiz and webpage from those e-mails, time spent on the quiz, information pages, and question of the week. Additionally, quiz and webpage users, number of sessions and engagement rate were recorded. Users are defined using cookies and unique identifiers which together result in the total number of new visitors to a certain page. However, when the same person accesses a webpage from a different device, a different location, or when they deleted their cookies, they may be recorded as a new user. Individuals who did not accept cookies are thus also not included in this data. Sessions can be interpreted as views of a certain page. One user can have multiple sessions on one page during a selected timeframe. Engagement rate is the proportion of sessions where the visitor was engaged. An engaged session is one where a user (1) stays on the webpage for more than 10 seconds, (2) views more than one page or screen, (3) completes >1 key event (e.g., a click on certain content). Information on video views and how many people opened the quiz, started and completed the quiz was also collected.

### Statistical analysis

For continuous variables, means and standard deviations (SD), or medians and interquartile ranges (IQR) are given, depending on the data distribution. Categorical data is described using counts and percentages. Participants who completed the entire study were compared with those that dropped out. This was done for continuous outcomes using unpaired t-tests or Mann-Whitney U tests depending on normality tests and visual inspection of the data-distribution. For binary outcomes, Chi^2^ tests were used. Further, ordered logistic regression was used for the outcome physical activity level.

Changes over time in knowledge and engagement in health behaviors were assessed using McNemar's tests, and linear, logistic, or ordinal mixed models with a random intercept. Age, gender, and educational level were always included as covariates. Demographic × time interactions were also explored but were removed from the model in case of non-significance. Changes over time in motivation for health behaviors were tested using ordinal mixed models. Again, age, gender, and educational level were included as covariates. For motivation for physical activity, baseline physical inactivity was also added to the model as covariate. Likewise, for motivation for a healthy diet, baseline adherence to the Mediterranean diet was added to the model. Time × demographic, or time × baseline Mediterranean diet adherence/physical inactivity interactions were also explored but removed in case of non-significance.

User experiences were compared between demographic groups and groups based on personal familiarity with dementia and awareness of dementia risk reduction at baseline. For this comparison, low and medium educated individuals were grouped together as the number of low-educated individuals was low (<5%). Individuals were also categorized by age (≤60 years old, 61–70 years old, > 70 years old). Comparative analyses were carried out using unpaired t-tests, and Mann-Whitney U tests for personal familiarity with dementia (due to highly skewed data and small group size). Multiple linear regression analyses were conducted to investigate whether engagement with the intervention (operationalized as the average score of the ratings of the seven weekly topics) was associated with (a) user ratings of the learning quality (i.e., ease of use, attractiveness and enjoyability, educational and motivating quality, meeting expectations) and (b) various outcomes (e.g., awareness of dementia risk reduction, overall knowledge of individual dementia risk/protective factors, LIBRA, BMI, cognitive activity, adherence to the Mediterranean diet, etc.). All tests were carried out two-sided with an alpha level of 0.05. All analyses were conducted with Stata 17.0 (StataCorp, College Station, TX, USA).

## Results

### Population characteristics

In total, 477 participants were recruited in November and December 2023. During that same period 1652 individuals registered for the e-learning, meaning that about 28.9% of them were included in the current study. Of the 477 participants who completed the baseline survey, 339 (71.1%) partook in the first follow-up survey immediately after the e-learning. 241 participants (50.5%) completed the entire study by also responding to the last survey, three months after completion of the e-learning. Detailed population characteristics are presented in Table 1.

**Table 1.** Population characteristics at baseline of the total sample and stratified by study completion.

Variable			Total sample (n = 477)	Completers (n = 241)	Dropouts (n = 236)	*p*
Age, mean (SD)			63 (12)	64 (11)	62 (12)	**0.027**
Age group, n (%)						**0.030**
	≤60 y		167 (35.0)	71 (29.5)	96 (40.7)	
	61–70 y		179 (37.5)	101 (41.9)	78 (33.1)	
	>70 y		131 (27.5)	69 (28.6)	62 (26.3)	
Female gender, n (%)			338 (70.9)	175 (72.6)	163 (69.1)	0.394
Educational level, n (%)						0.342
	Low		19 (4.0)	8 (3.3)	11 (4.7)	
	Medium		184 (38.6)	87 (36.1)	97 (41.1)	
	High		274 (57.4)	146 (60.6)	128 (54.2)	
Personally familiar with dementia, n (%)			432 (90.6)	218 (90.5)	214 (90.7)	0.934
Modifiable dementia risk factors						
	Current smoker, n (%)		26 (5.5)	7 (2.9)	19 (8.1)	**0**.**013**
	BMI, mean (SD)		25.3 (4.3)	24.9 (4.1)	25.7 (4.5)	**0**.**050**
	Physical activity level*, n (%)					0.203
		Inactive	72 (15.1)	31 (12.9)	41 (17.4)	
		Moderately inactive	112 (23.5)	56 (23.2)	56 (23.7)	
		Moderately active	135 (28.3)	70 (29.1)	65 (27.5)	
		Active	158 (33.1)	84 (34.9)	74 (31.4)	
	Average alcohol units/week, median (IQR)		1.1 (0.3–6)	1.0 (0.3–6)	1.0 (0.3–6)	0.955
	Cognitive activity score, mean (SD)		22.0 (5.3)	22.3 (5.4)	21.8 (5.2)	0.329
	Adherence to Mediterranean diet †, mean (SD)		6.8 (1.9)	6.8 (1.9)	6.7 (2.0)	0.570
	Social contact satisfaction †, mean (SD)		7.4 (1.8)	7.5 (1.7)	7.2 (2.0)	**0**.**034**
	Sleep quality †, mean (SD)		6.7 (1.9)	6.8 (1.9)	6.6 (1.9)	0.238
	Mood †, mean (SD)		7.6 (1.6)	7.7 (1.6)	7.5 (1.7)	0.189
	Hypertension, n (%)		141 (30.0)	68 (28.5)	73 (31.6)	0.456
	Dyslipidemia, n (%)		170 (36.9)	88 (37.3)	82 (36.4)	0.851
	Diabetes, n (%)		31 (6.5)	15 (6.2)	16 (6.8)	0.787
	Coronary heart disease, n (%)		63 (13.4)	26 (10.9)	37 (16.0)	0.102
	Chronic kidney disease, n (%)		13 (2.7)	5 (2.1)	8 (3.4)	0.365
LIBRA score, mean (SD)			0.5 (2.5)	0.2 (2.5)	0.7 (2.4)	**0.049**
Aware of dementia risk reduction, n (%)			356 (74.6)	178 (73.9)	178 (75.4)	0.154
Knowledge score, mean (SD)			41 (8)	41 (7)	40 (8)	**0.029**
Motivation						
	Healthy diet ‡, mean (SD)		2.8 (1.6)	2.7 (1.6)	2.8 (1.6)	0.625
	Physical activity ‡, mean (SD)		2.8 (1.5)	2.6 (1.5)	2.9 (1.5)	**0**.**020**

Theoretical range LIBRA score: −5.9 to +12.7, theoretical range knowledge score: 0 to 64. Missing: dyslipidemia (n = 16), CHD (n = 7), hypertension (n = 7), diabetes (n = 2), CKD (n = 3). BMI: body mass index; SD: standard deviation; IQR: interquartile range; LIBRA: LIfestyle for BRAin health

*Tested using ordered logistic regression.

† rated on a scale between 0–10.

‡ Surveyed as which of the following statements is most applicable. Five statements indicative of increasing levels of motivation were listed. These were analyzed as values ranging between 1–5. Numbers show mean values for each group. Comparative analysis was done using ordered logistic regression.

### Completers versus dropouts

Certain differences between individuals who completed the entire study (n = 241) and those who dropped out (n = 236) were noted. Individuals who dropped out were on average younger, more often smokers, had a higher BMI, were less satisfied with their social contacts, had a higher modifiable dementia risk (LIBRA score), and less knowledge about modifiable dementia risk/protective factors at baseline. Their self-reported motivation for physical activity was, however, higher.

### Knowledge of dementia risk reduction

**Figure 5.**
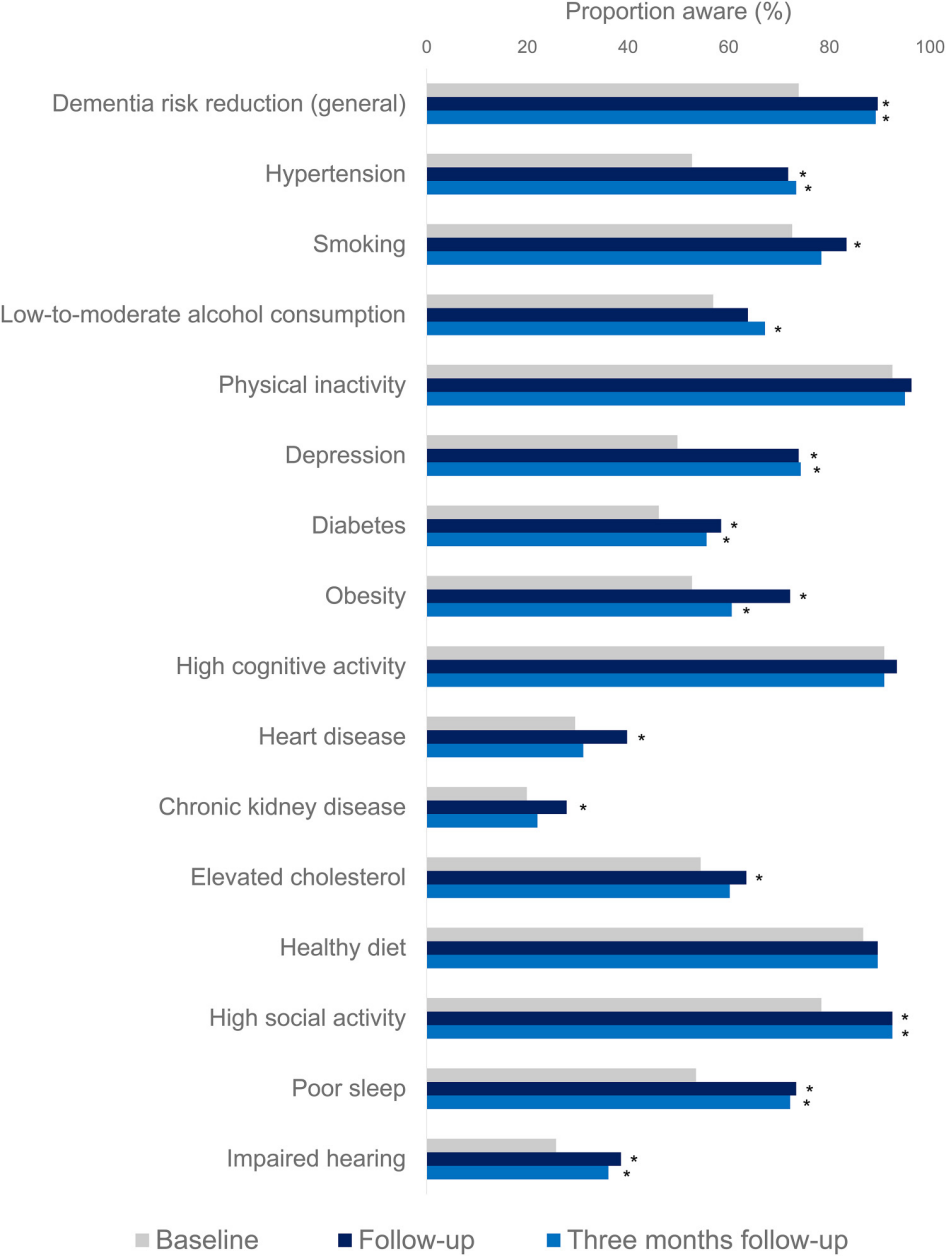
Awareness of dementia risk reduction in general and individual dementia risk/protective factors over time. Sample limited to participants who completed the entire study (n = 241). Tested using McNemar's tests (*: significant difference compared to baseline).

At baseline, 356 participants (74.6%) of the total sample (n = 477) were already aware of the potential for dementia risk reduction. Immediately after the e-learning (compared to baseline), more individuals were aware of dementia risk reduction after the e-learning (231 (75.0%) versus 277 (89.9%), *p* < 0.001, Figure 5). Linear mixed model analysis confirmed these findings (awareness: b_immediate _= 1.50, *p* < 0.001 and b_3 months _= 1.43, *p* < 0.001) but added that younger individuals were already significantly more aware at baseline (b_age _= −0.03, *p* = 0.021). There were no differences in change in awareness over time between demographic groups.

Awareness of most individual risk/protective factors also increased by follow-up (Figure 5; n = 241). Overall knowledge of modifiable risk and protective factors at baseline was higher among highly educated individuals (b = 1.98, *p* = 0.004, compared to low or medium educated individuals), and younger individuals (b_age _= −0.06, *p* = 0.035). Knowledge of modifiable risk and protective factors increased over time (b_immediate _= 5.11, *p* = 0.034 and b_3 months _= 5.59, *p* = 0.042). By immediate follow-up, knowledge had increased significantly more among women compared to men (b_gender × time _= 1.65, *p* = 0.046) although this interaction was no longer observed after three months. Three months after completion of the e-learning, 215 out of 241 (89.0%) were aware of the possibility of dementia risk reduction, which was still significantly higher compared to baseline (*p* < 0.001, Figure 5).

### Health behaviors

At baseline (n = 477), self-rated adherence to the Mediterranean diet was higher among women (b = 0.62, *p* < 0.001, older individuals (b_age _= 0.02, *p* = 0.020), and highly educated individuals (b = 0.47, *p* = 0.003). Adherence to the Mediterranean diet also increased over time (b_immediate _= 0.27, *p* = 0.002 and b_3 months _= 0.32, *p* = 0.001). At baseline, weekly alcohol consumption was lower among women (b = −2.17, *p* < 0.001). No overall effect of time on alcohol consumption was observed, yet, at three months follow-up, alcohol consumption was significantly decreased in women compared to men (b = −1.21, *p* = 0.008). Over all time points, the gender × time interaction was indeed significant (*p* = 0.015). At baseline, cognitive activity scores were higher among women (b = 1.94, *p* < 0.001), older individuals (b_age _= 0.12, *p* < 0.001), and highly educated individuals (b = 1.93, *p* < 0.001). They also appeared to increase over time (b_immediate _= 0.89, *p* < 0.001 and b_3 months _= 0.87, *p* < 0.001). Yet, when adding demographic × time interactions to the model, an overall effect of time was no longer observed (b_immediate _= 0.96, *p* = 0.443 and b_3 months _= 2.59, *p* = 0.071). Instead, a time × gender interaction becomes apparent: women increased their cognitive activity scores more compared to men at immediate follow-up (b_gender × time _= 0.85, *p* = 0.048). Additionally, social contact satisfaction improved by completion of the e-learning (b_immediate _= 0.15, *p* = 0.018) although this difference was no longer observed after three months (b_3 months _= 0.06, *p* = 0.403). Women were more satisfied with their social contacts at baseline (b = 0.62, *p* < 0.001). Physical activity level was lower at baseline among older individuals (b_age _= −0.05, *p* < 0.001) and physical activity level appeared to decrease over time (b_immediate _= −1.00, *p* < 0.001 and b_3 months _= −0.59, *p* = 0.001). However, when adding demographic × time interactions to the model, there was no significant overall effect of time but significant interactions between time × educational level, and time × age were observed. At immediate follow-up, physical activity level had decreased more among older compared to younger individuals (b_age × time _= −0.03, *p* = 0.021), whereas after three months follow-up highly educated individuals increased their physical activity level compared to low- or medium educated individuals (b_education × time _= 0.91, *p* = 0.017). Over all time points, the age × time interaction was also significant (*p* = 0.026) but the educational level × time interaction was not (*p* = 0.055). BMI was significantly lower at baseline among high educated individuals (b = −1.13, *p* = 0.004) and younger individuals (b_age _= −0.04, *p* = 0.028). BMI did not change over time. There were no differences in self-rated sleep quality or mood between demographic groups, nor over time. LIBRA at baseline was significantly lower among higher educated individuals (b_high educated _= −0.59, *p* = 0.008), among women (b = −1.03, *p* < 0.001), and among younger individuals (b_age _= 0.03, *p* = 0.005). LIBRA did not change over time overall. However, LIBRA was significantly lower after three months among highly educated individuals compared to low- or medium educated individuals (b_time × education _= −0.39, *p* = 0.044). The number of participants that smoked was very limited (n_baseline _= 26 (5.5%), n_3 months _= 7 (2.9%)). Therefore, no analyses on smoking behavior were ran.

The majority of individuals with a history of hypertension (n = 84), dyslipidemia (n = 111), or diabetes (n = 17) at baseline reported to have taken action to either lower their blood pressure, improve their blood lipid levels, or manage their glycaemia better, by three months follow-up (Table 2).

**Table 2.** Self-reported actions to manage hypertension, dyslipidemia, or diabetes three months after completion of the e-learning.

	History at baseline of		
	Hypertension, n (%)	Dyslipidemia, n (%)	Diabetes, n (%)
n_total_	84	111	17
Took action	55 (65.5)	70 (63.1)	12 (70.6)
Wants to take action in the future	4 (4.8)	6 (5.4)	0 (0)
Did not take action, not planning to	6 (7.1)	14 (12.6)	0 (0)
Did not take action, already well-managed	19 (22.6)	21 (18.9)	5 (29.4)

Missing: actions to manage for hypertension (n = 8), dyslipidemia (n = 3), diabetes (n = 11).

### Motivation for health behaviors

Motivation for physical activity was higher at immediate follow-up compared to baseline (b = 3.14, *p* = 0.003), and this increase was larger among younger (b_age × time _= −0.04, *p* = 0.005) and low-to-medium educated individuals (b_highly educated _= −0.67, *p* = 0.043). At three months follow-up, however, there was no difference in motivation for physical activity compared to baseline. Motivation for a healthy diet, smoking cessation, or reducing alcohol consumption did not change over time. Demographic differences in baseline motivation were also observed. Detailed results on motivation for health behaviors can be consulted in Supplemental Material 4.

### User experience

316 participants rated the e-learning's overall quality. Herein, it scored 8.2 out of 10 (SD = 1.8) for ease of use, 7.7 out of 10 (SD = 1.8) for attractiveness and enjoyability, and 7.6 out of 10 (SD = 1.8) for the overall perceived educational and motivating quality. In addition, participants rated the e-learning a 7.0 out of 10 (SD = 2.3) for meeting their expectations. Women consistently rated the e-learning higher on all four of these dimensions (all *p* < 0.050). Individuals between 61 and 70 years old rated the e-learning higher for meeting their expectations (7.3 versus 6.6, *p* = 0.034), attractiveness and enjoyability (8.0 versus 7.4, *p* = 0.028), and educational and motivating quality (7.9 versus 7.2, *p* = 0.010), compared to older individuals (>70 years old). The youngest individuals (≤60 years old) appeared to be in between these two groups in terms of their ratings but did not differ significantly from the older age groups. There were no differences in these ratings based on educational level, personal familiarity with dementia, or baseline awareness of dementia risk reduction.

**Figure 6.**
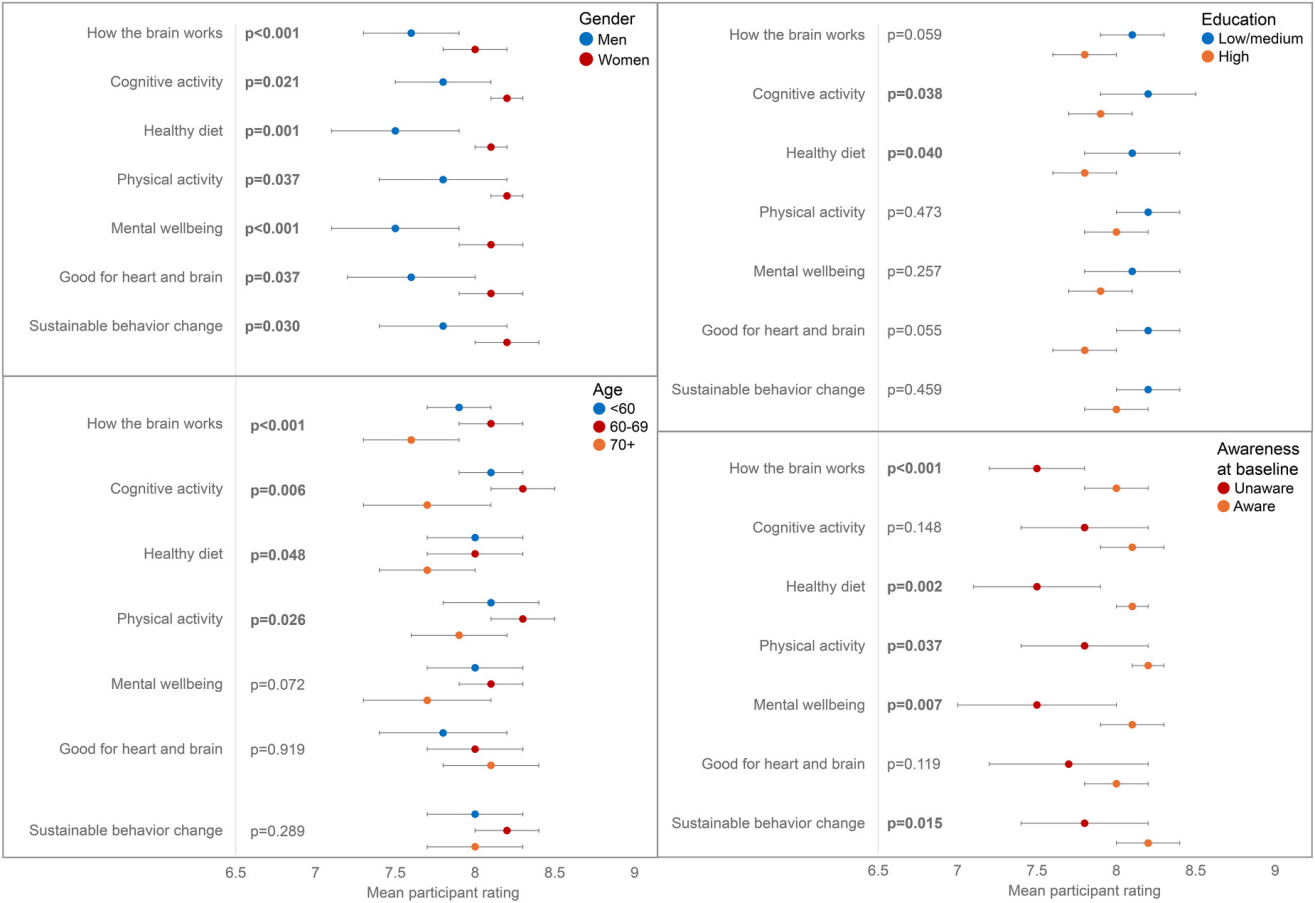
E-learning weekly theme ratings (on a scale of 0–10) by demographic groups and awareness of dementia risk reduction at baseline. Compared using unpaired t-test. p-values for age are those comparing 61–70 with >70, all other comparisons between age groups were non-significant.

All individual weekly topics were rated similarly, ranging between 7.9 and 8.1 out of 10. Again, women consistently rated all topics higher than men did (all *p* < 0.050, Figure 6). Most topics tended to be rated highest by the 61–70-year-old respondents and the lowest by the respondents >70 years old (Figure 6). Low-and-medium-educated individuals rated the themes, ‘Cognitive activity’ and ‘Healthy diet’, higher than the highly educated individuals. Individuals who already were aware of dementia risk reduction at baseline, rated all themes besides ‘Cognitive activity’ and ‘Good for the heart, good for the brain’ higher than unaware individuals (Figure 6). There were no differences in the e-learning ratings by personal familiarity with dementia.

### Engagement with the e-learning

**Figure 7.**
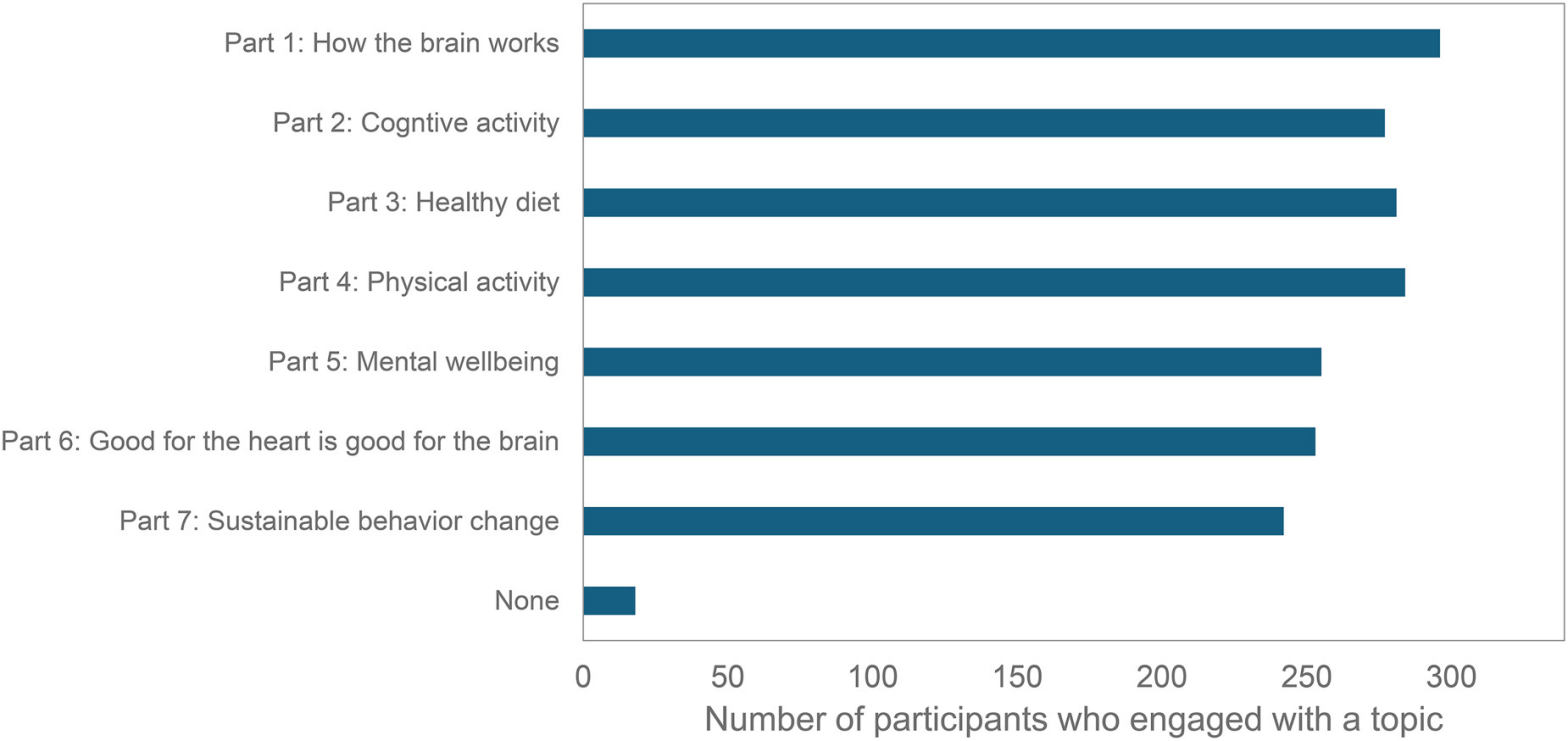
Self-reported engagement with the e-learning topics (n_total _= 339).

Self-reported engagement with the e-learning topics and materials overall was high but appeared to decline somewhat over the seven-week time period (Figure 7; n = 339). Of all participants who completed the survey immediately after the e-learning, 296 (87.3%) reported to have engaged with the first part of the e-learning, compared to 242 (71.4%) who engaged with the last part. Results from multiple linear regression analyses showed that a higher mean score on engagement was associated with higher scores on each of the four dimensions of learning quality, even after adjustment for age, gender and educational level (all *p* < 0.050). No significant associations were found between engagement scores and various outcome measurements (e.g., awareness of dementia risk reduction, overall knowledge of individual dementia risk/protective factors, LIBRA, BMI, cognitive activity, adherence to the Mediterranean diet, etc.).

Usage data demonstrated similar findings to the results reported above for self-reported engagement. The number of participants who opened the weekly e-learning e-mail declined somewhat over time (from 96.0% to 84.1%) and tended to be higher among participants compared to non-participating users, although not consistently (Supplemental Material 5, Supplemental Table 1 and Supplemental Material 5, Supplemental Figure 1). Yet, clicks from the first e-mail to the quiz started at 49.5%, increased to 71.3% in the 3^rd^ e-mail about a healthy diet, and then slowly declined to 60.6% in the last e-mail.

Quiz usage data demonstrated that of those participants opening the quiz, about 40–47% also started answering quiz items, and about 38–44% completed the quiz. For non-participating users (all users since implementation of quiz), 50–52% started answering quiz items, and about 40–44% completed the quiz. Google Analytics-based data on engagement with the quiz and webpage among regular users during the time period of the study, again showed a decline in users and sessions over time (Supplemental Material 5, Supplemental Table 3). The quiz was most often engaged with total users ranging from 8432 users for Theme 1 to 2609 users for Theme 7. At the beginning of the course, the e-training webpages were visited around half as often as the quiz. By the end of the course, the e-training webpages were visited 25% as often as the quiz. When individuals visited the quiz or webpage, engagement percentage was consistently very high (>80%, Supplemental Material 5, Supplemental Table 3), and about 11–40% of users watched the educational video, again with the proportion declining over time.

## Discussion

In this single-arm pre-post intervention study evaluating a free, online e-learning about dementia risk reduction, significant positive changes over time in terms of knowledge of modifiable risk and protective factors for dementia, motivation for physical activity, and several lifestyle factors were observed, some of which remained up to three months after e-learning completion. Moreover, user experiences were very positive, with women and individuals aged 60–69 years old rating the e-learning highest.

Individuals who registered for the e-learning and agreed to participant in this pre-post study showed improvements over time in their knowledge of dementia risk reduction, motivation, and multiple lifestyle factors.^42^ Specifically, improvements were seen in motivation for physical activity, adherence to a Mediterranean diet, social contact satisfaction, cognitive activity levels and alcohol consumption in women, and physical activity levels and the LIBRA score in highly educated individuals. Moreover, improvements in physical activity levels and the LIBRA score in highly educated individuals, and knowledge and Mediterranean diet adherence in the whole sample were still present three months after completion of the e-learning. Although improvements in lifestyle factors were small, these small changes may translate into tangible health benefits when implemented in large populations.^43^ Moreover, given this cheap, easy-access, short, low-intensity intervention, implementation in large populations is very attainable. These findings are in line with outcomes from the dementia prevention MOOC of the University of Tasmania.^20^ This mixed-methods study found that diet, physical activity and cognitive activity were the most commonly reported behavior changes.

The e-learning was rated very positively. Women consistently rated the e-learning higher than men which was also seen for the dementia prevention MOOC of the University of Tasmania.^20^ Women also showed larger increases in knowledge immediately after completion of the e-learning. Interestingly, the e-learning was rated highest by people between 60–69 years old, who rated it significantly better than those aged ≥ 70 years old. This age group might comprise a real window of opportunity, constituting of individuals who are transitioning from one life stage to the next (e.g., via retirement), are well-acquainted with technology and the internet, and may be more familiar and aware of aging-related conditions such as dementia.^21,44,45^ Further, engagement with the online course was relatively high which is indicative of good usability.^46–48^ It appears that once individuals registered, and especially when they decided to view the e-learning webpages, they were very engaged with its content. Conversely, reaching individuals, especially low-educated, with less affinity for health and health promotion, to register for the online course may be the most important barrier to overcome.

Thanks to collaboration with societal stakeholder Alzheimer Nederland and their established media channels (i.e., in 2021 Alzheimer Nederland informed >100.000 people about dementia prevention/brain health through their brochures, newsletters and website), the inclusion target was reached within two months. Participants were community-dwelling individuals who voluntarily registered for this Dutch e-learning, suggesting an affinity for dementia and health promotion. A considerable 28.9% of individuals who registered for the e-learning was included in the study. These individuals already were very knowledgeable about dementia risk reduction at baseline, with 74.6% being aware of the potential for dementia risk reduction, which is much higher than other survey studies in other populations.^5–7,19^ Participants were mostly women and highly educated, which is a common observation for e-learning enrollees, and online health information seekers in general.^20,49,50^ This limits external validity and, consequently, these results cannot speak to potential changes in other populations (e.g., lower educated, men, unhealthier lifestyle), when they would be provided with access to this online course. Moreover, these results indicate that we still failed to recruit and reach those individuals that could benefit most from this health information: low educated individuals who are unaware of dementia risk reduction. In addition, individuals who are more at-risk for dementia based on lifestyle and cardiovascular factors are more often underrepresented in these kind of studies. More knowledge is urgently needed on how to reach those underrepresented individuals effectively, which is one of the research questions for which the Netherlands Dementia Prevention Initiative (NDPI) aims to provide an answer to in the coming years.^51^ In this national project, we will develop and evaluate several inclusive engagement/sampling strategies (e.g., focus groups, co-creation techniques) and tools in order to include underrepresented high-risk groups in future dementia risk reduction interventions.

Physical activity level increased after three months in highly educated individuals compared to low- or medium educated individuals. Conversely, at immediate follow-up physical activity levels decreased in older compared to younger individuals. There is no clear explanation for these observations. However, the finding that highly educated individuals improved more over time appears to be in line with multiple other results (e.g., LIBRA improving more in highly educated individuals; women reducing their alcohol consumption more compared to men, and increasing their cognitive activity levels more compared to men) collectively suggesting that often the individuals that already lived a more ‘brain-healthy’ lifestyle tended to be the ones that also showed more improvement over the course of this e-learning. This is exactly the opposite of what we aim for, which is providing support for those that need it most. As such, not only information on reaching those individuals that could benefit most is urgently needed, but also knowledge on developing effective interventions for these groups. Future research should focus on the reasons why people dropped out or engaged less with the e-learning. It is important to follow these individuals up (e.g., telephonic interview) in order to find the underlying cause of why people do not engage with these kind of interventions. This will provide an overview of what aspects they may like from the e-learning and could potentially uncover barriers that could be addressed in future studies. Engagement with these kind of interventions could also be increased by involving health care professionals (as a form of ‘prescription’ to the e-learning), by creating a participant community (e.g., chat-function to share tips, online forum, leaderboard), or by recruiting individuals from certain community groups where more at-risk individuals may be part of (e.g., individuals with a migration background) through closely-involved key persons such as community group leaders/gatekeepers. These kind of engagement and recruitment strategies will be investigated by the NDPI project.

Strengths of the current study include its sufficiently large sample size to conduct analyses with enough power and the three-month follow-up assessment point to investigate sustainability of changes during the e-learning. Importantly, we tested for differences over time in the outcomes of interest which has major benefits compared to only surveying perceived changes over time at follow-up, which is commonly done when evaluating health promotion tools.^20,52^ The usage data further allowed us to get an idea on comparability between participants and non-participating users which can serve as an indicator of internal validity. Differences in e-learning usage between participants and non-participating users were observed but these were relatively small, suggesting that participants may be fairly representative of all users of this e-learning in terms of usage.

This study also had certain limitations. Importantly, this is a pre-post intervention study without a control group. This design was chosen as defining an appropriate and feasible (active) control group was difficult. With this design, we also aimed to include more individuals who were more representative for the general population. However, because of this, we cannot attribute the observed changes over time solely to the e-learning. As discussed previously, external validity is reduced due to the specific sample of highly educated individuals who already were very knowledgeable about dementia risk reduction at baseline. Moreover, follow-up surveys were not completed by everyone, further narrowing down the sample. Indeed, individuals who completed the study differed from individuals who dropped out, for example in age, smoking status, and knowledge about dementia risk reduction. Individuals who knew less about the topic dropped out more often which is the opposite of what we aim for but also a common finding with health promotion interventions.^53^ We attempted to use validated questionnaires to assess all constructs of interest, but this was not always possible as these were sometimes simply not available or would be too time-consuming for the participants when filling in the surveys (max. 15 min/survey). Therefore, multiple custom items were also used, with unknown construct validity (e.g., adherence to a Mediterranean diet, mood, social contact satisfaction).

Taken together, participation in this low-intensity, free e-learning program was associated with increases in knowledge of dementia risk reduction and small improvements in multiple lifestyle factors related to dementia risk, some of which remained present three months after e-learning completion. The e-learning can easily be implemented as a stand-alone tool or as part of larger dementia risk reduction initiatives. Future research should focus on reaching those individuals that could benefit most from the information/health advice provided by this e-learning.

## Supplemental Material

sj-docx-1-alz-10.1177_13872877241309112 - Supplemental material for Development and evaluation of a free e-learning program on dementia risk reduction for the general public: A pre-post study Supplemental material, sj-docx-1-alz-10.1177_13872877241309112 for Development and evaluation of a free e-learning program on dementia risk reduction for the general public: A pre-post study by Stephanie Van Asbroeck, Sophie CPM Wimmers, Martin PJ van Boxtel and Rob BM Groot Zwaaftink, Vera Otten, Dinant Bekkenkamp, Sebastian Köhler, Kay Deckers in Journal of Alzheimer's Disease

sj-docx-2-alz-10.1177_13872877241309112 - Supplemental material for Development and evaluation of a free e-learning program on dementia risk reduction for the general public: A pre-post study Supplemental material, sj-docx-2-alz-10.1177_13872877241309112 for Development and evaluation of a free e-learning program on dementia risk reduction for the general public: A pre-post study by Stephanie Van Asbroeck, Sophie CPM Wimmers, Martin PJ van Boxtel and Rob BM Groot Zwaaftink, Vera Otten, Dinant Bekkenkamp, Sebastian Köhler, Kay Deckers in Journal of Alzheimer's Disease

sj-docx-3-alz-10.1177_13872877241309112 - Supplemental material for Development and evaluation of a free e-learning program on dementia risk reduction for the general public: A pre-post study Supplemental material, sj-docx-3-alz-10.1177_13872877241309112 for Development and evaluation of a free e-learning program on dementia risk reduction for the general public: A pre-post study by Stephanie Van Asbroeck, Sophie CPM Wimmers, Martin PJ van Boxtel and Rob BM Groot Zwaaftink, Vera Otten, Dinant Bekkenkamp, Sebastian Köhler, Kay Deckers in Journal of Alzheimer's Disease

sj-docx-4-alz-10.1177_13872877241309112 - Supplemental material for Development and evaluation of a free e-learning program on dementia risk reduction for the general public: A pre-post study Supplemental material, sj-docx-4-alz-10.1177_13872877241309112 for Development and evaluation of a free e-learning program on dementia risk reduction for the general public: A pre-post study by Stephanie Van Asbroeck, Sophie CPM Wimmers, Martin PJ van Boxtel and Rob BM Groot Zwaaftink, Vera Otten, Dinant Bekkenkamp, Sebastian Köhler, Kay Deckers in Journal of Alzheimer's Disease

sj-docx-5-alz-10.1177_13872877241309112 - Supplemental material for Development and evaluation of a free e-learning program on dementia risk reduction for the general public: A pre-post study Supplemental material, sj-docx-5-alz-10.1177_13872877241309112 for Development and evaluation of a free e-learning program on dementia risk reduction for the general public: A pre-post study by Stephanie Van Asbroeck, Sophie CPM Wimmers, Martin PJ van Boxtel and Rob BM Groot Zwaaftink, Vera Otten, Dinant Bekkenkamp, Sebastian Köhler, Kay Deckers in Journal of Alzheimer's Disease
