# Supplementary material for: Development and evaluation of a free e-learning program on dementia risk reduction for the general public: A pre-post study
Source: J Alzheimers Dis. 2025 Jan 10;103(4):1075–89. doi: 10.1177/13872877241309112 (PMC12231795; doi:10.1177/13872877241309112)
Supplement: sj-docx-1-alz-10.1177_13872877241309112 - Supplemental material for Development and evaluation of a free e-learning program on dementia risk reduction for the general public: A pre-post study [file sj-docx-1-alz-10.1177_13872877241309112.docx]

**Supplemental Material 1: Needs assessment**

A needs assessment was carried out by sending an online survey to previous users of the pre-existing e-learning via e-newsletter (at that moment 81.194 subscribers). 172 individuals completed each of the survey questions. About 76% of the respondents identified as female. Most individuals were between 50-89 years old, predominantly between 60-79 years old, and most were highly educated.

The most interesting themes according to respondents were ‘How the brain works’, and the theme on a healthy diet, followed by those on physical activity and cognitive activity. Survey responses indicated that the quiz was by far the most popular part of the weekly e-learning content. This was followed by the webpage, and the weekly challenge. Individuals generally did not want a very thorough, extensive course. Instead, most individuals indicated they prefer to spend between 5-15 minutes on each weekly theme. As such, respondents typically indicated they liked the concise format, although some people also wanted more in-depth scientific background, about pre-existing themes (most commonly physical activity, ‘How the brain works’, and building new habits). New themes were also suggested, such as sleep, new research that emerges, and what is normal cognitive aging versus what is not. Importantly, about 42% of respondents indicated they would like more video content, and 29% indicated they would like more images. About 19% also indicated they would like a podcast. Lastly, the majority of respondents indicated that they did not have a desire to receive a certificate of completion at the end of the e-learning.
