# Supplementary material for: Development and evaluation of a free e-learning program on dementia risk reduction for the general public: A pre-post study
Source: J Alzheimers Dis. 2025 Jan 10;103(4):1075–89. doi: 10.1177/13872877241309112 (PMC12231795; doi:10.1177/13872877241309112)
Supplement: sj-docx-2-alz-10.1177_13872877241309112 - Supplemental material for Development and evaluation of a free e-learning program on dementia risk reduction for the general public: A pre-post study [file sj-docx-2-alz-10.1177_13872877241309112.docx]

**Supplemental Material 2. Selection of the e-learning webpage content**

Part 1: Your fit brain

**
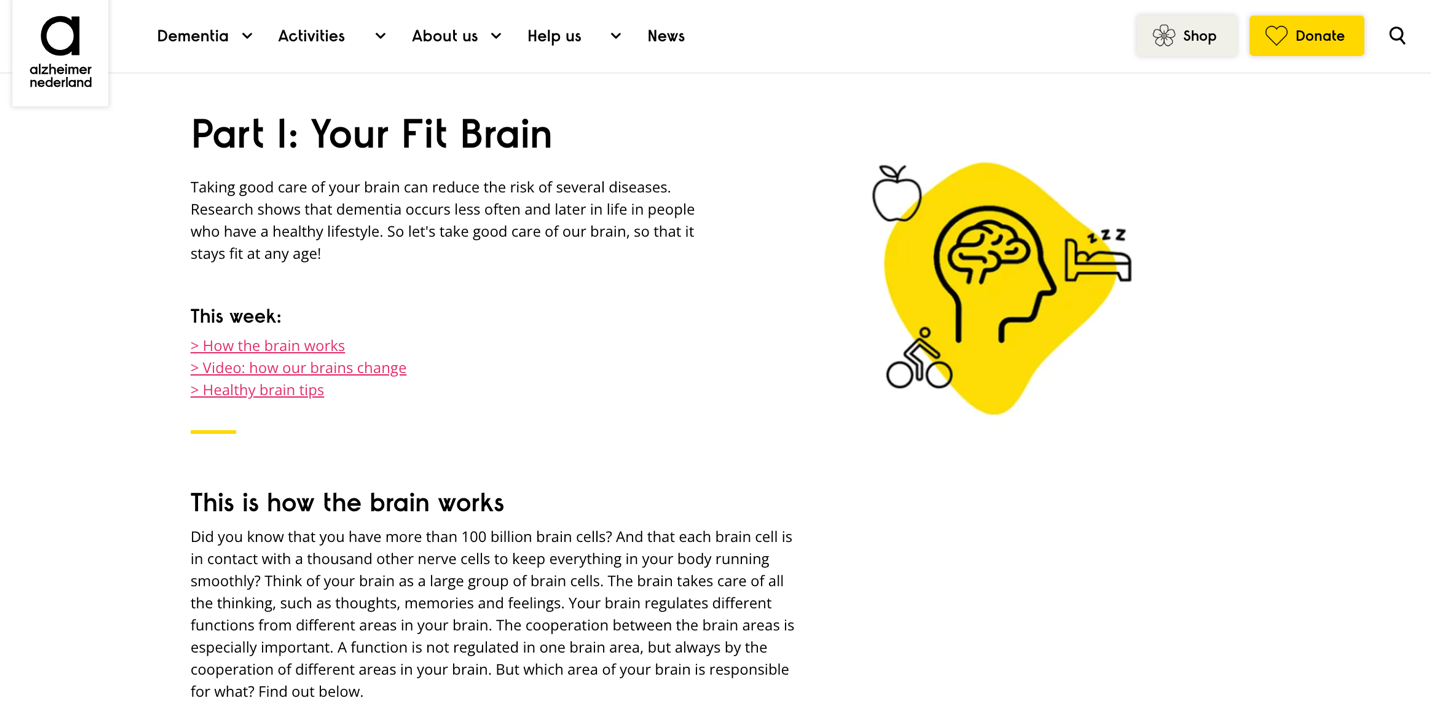
**

**
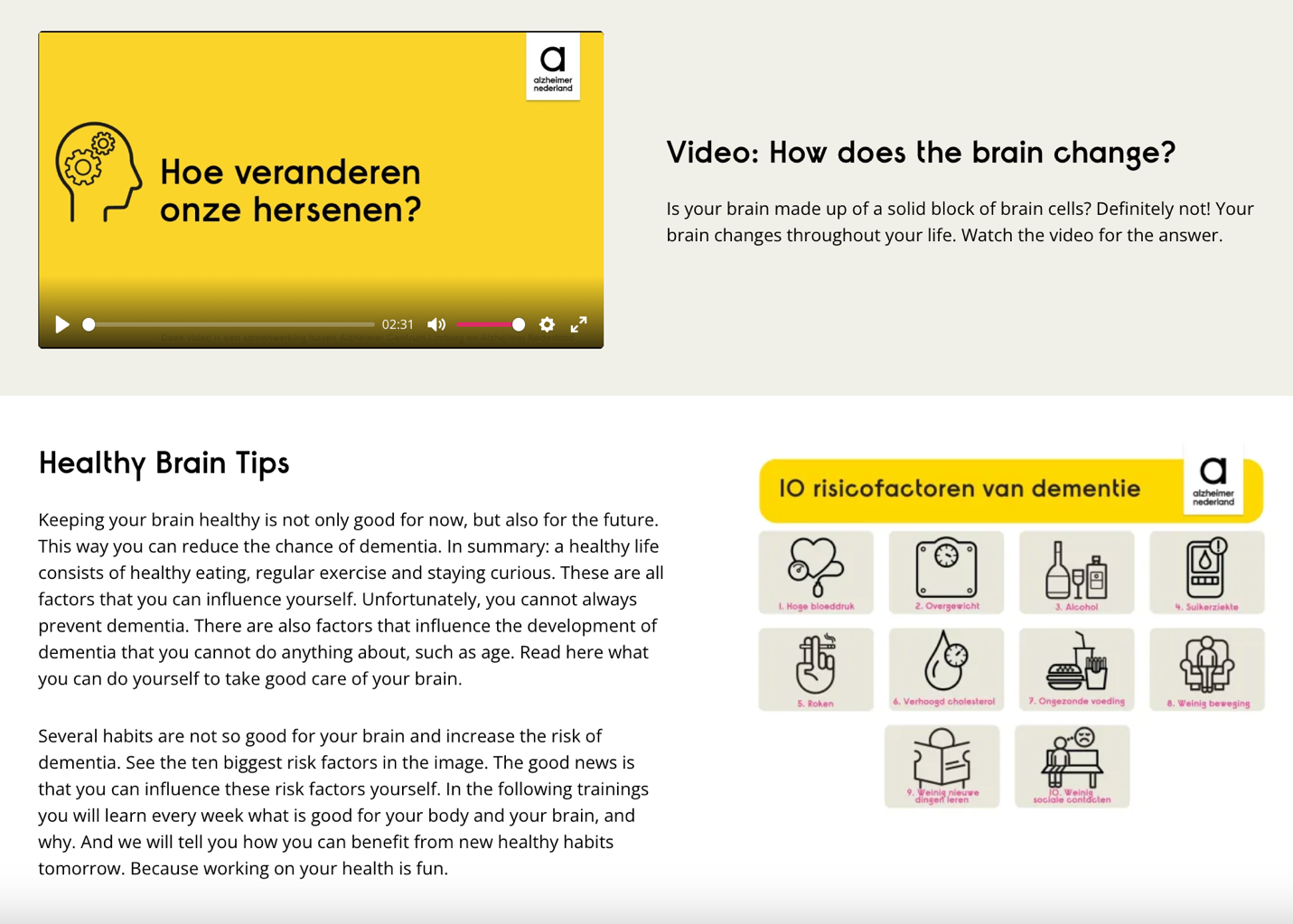
**

Part 2: Challenge your brain

**
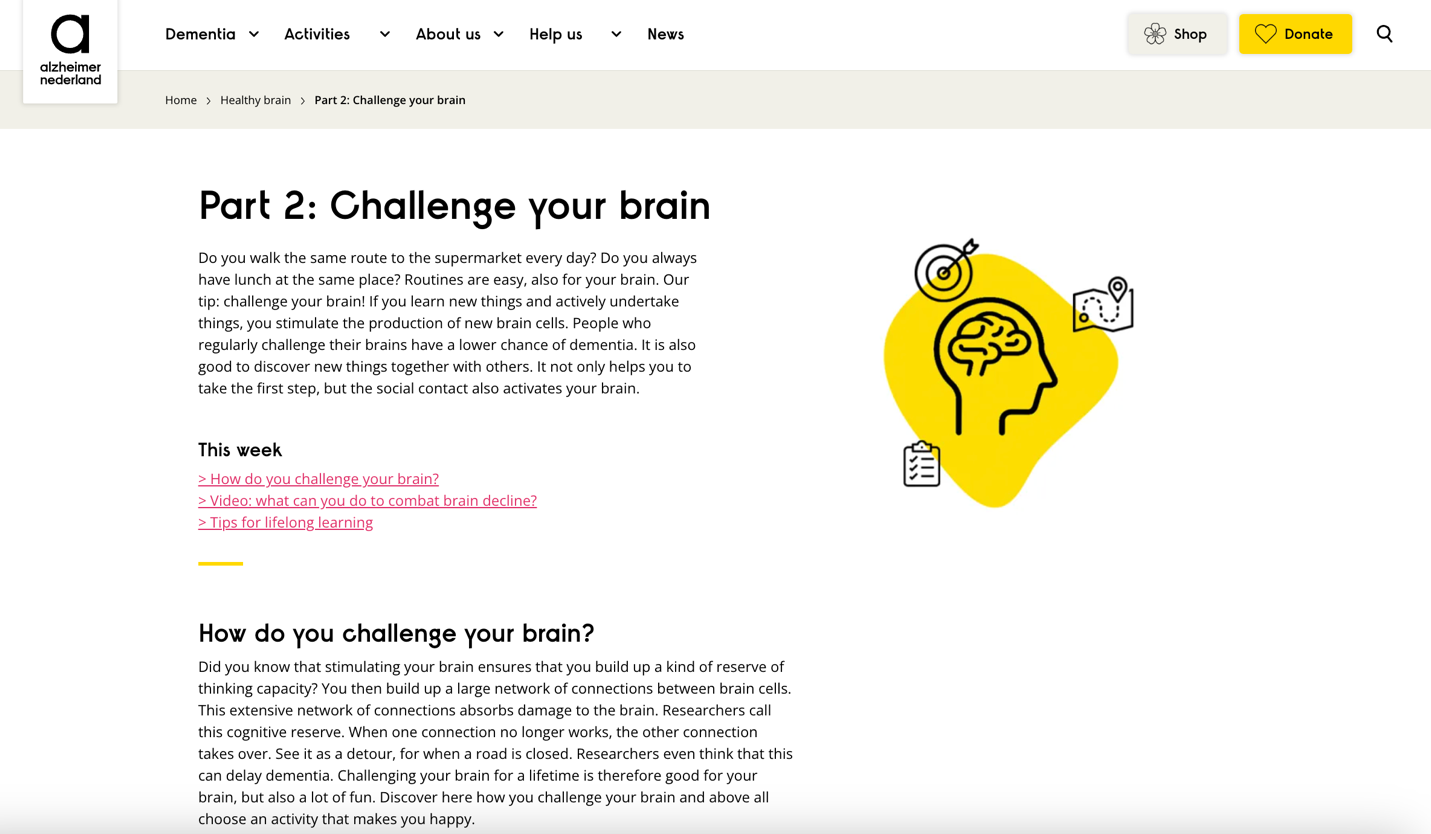
**

**
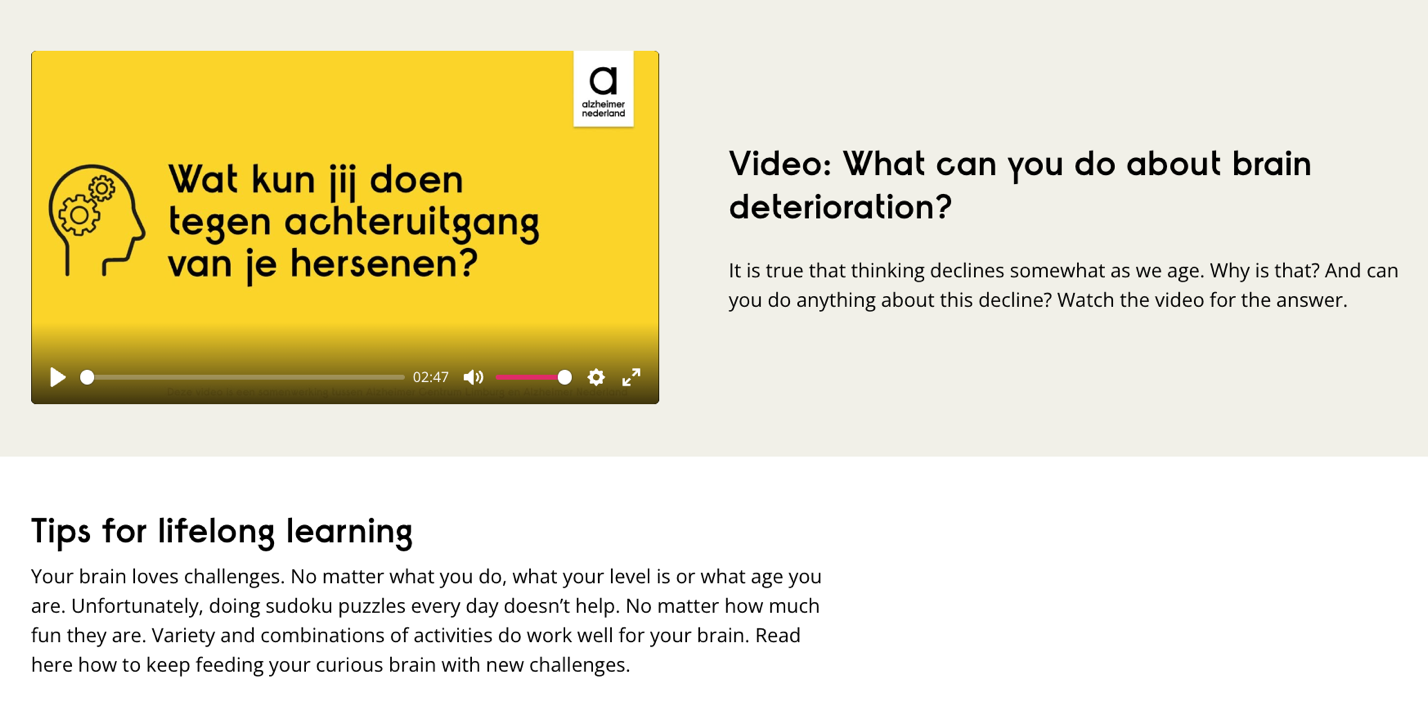
**

Part 3: Eat your brain healthy

**
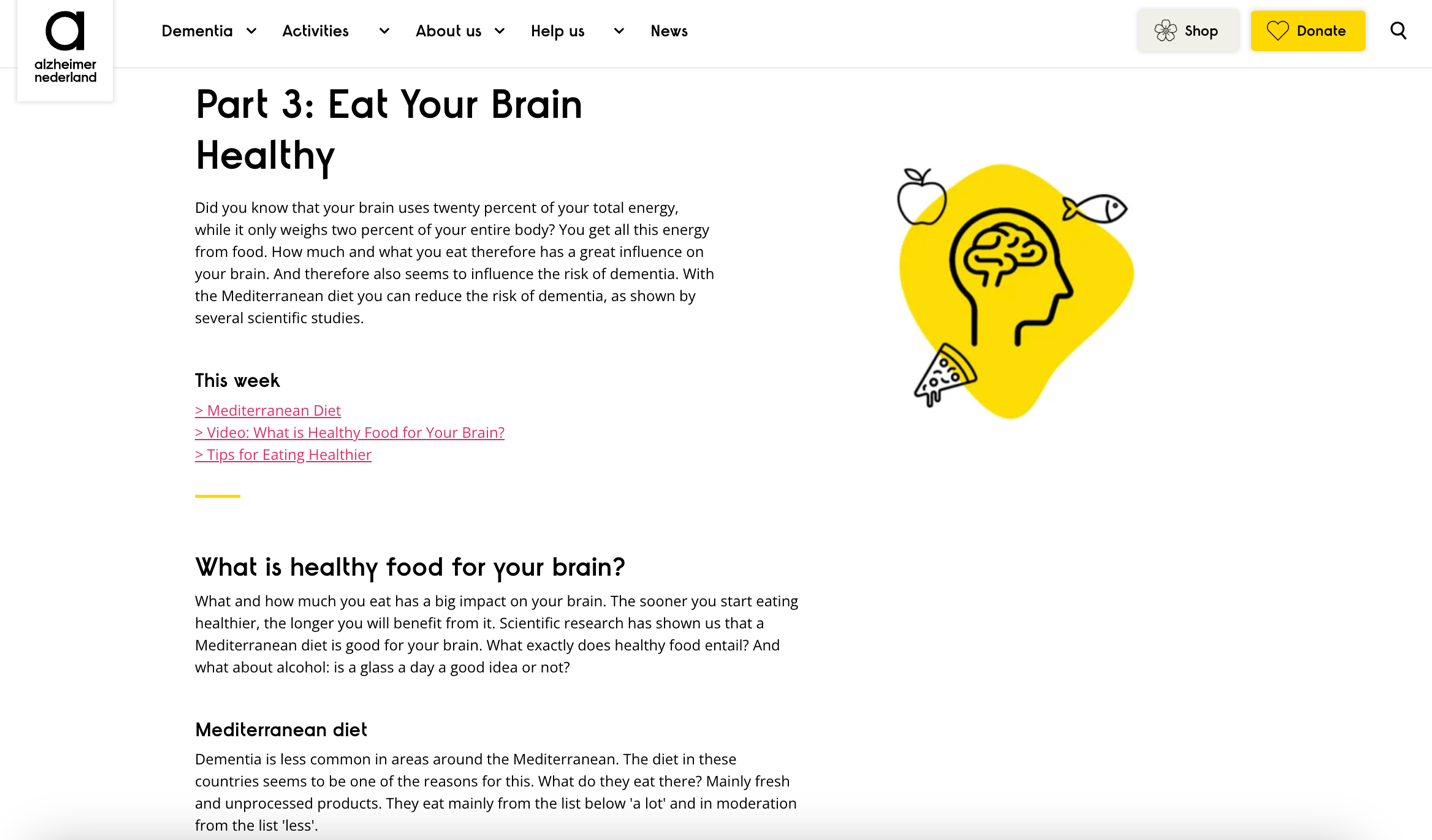
**

Part 4: Move your brain healthy

**
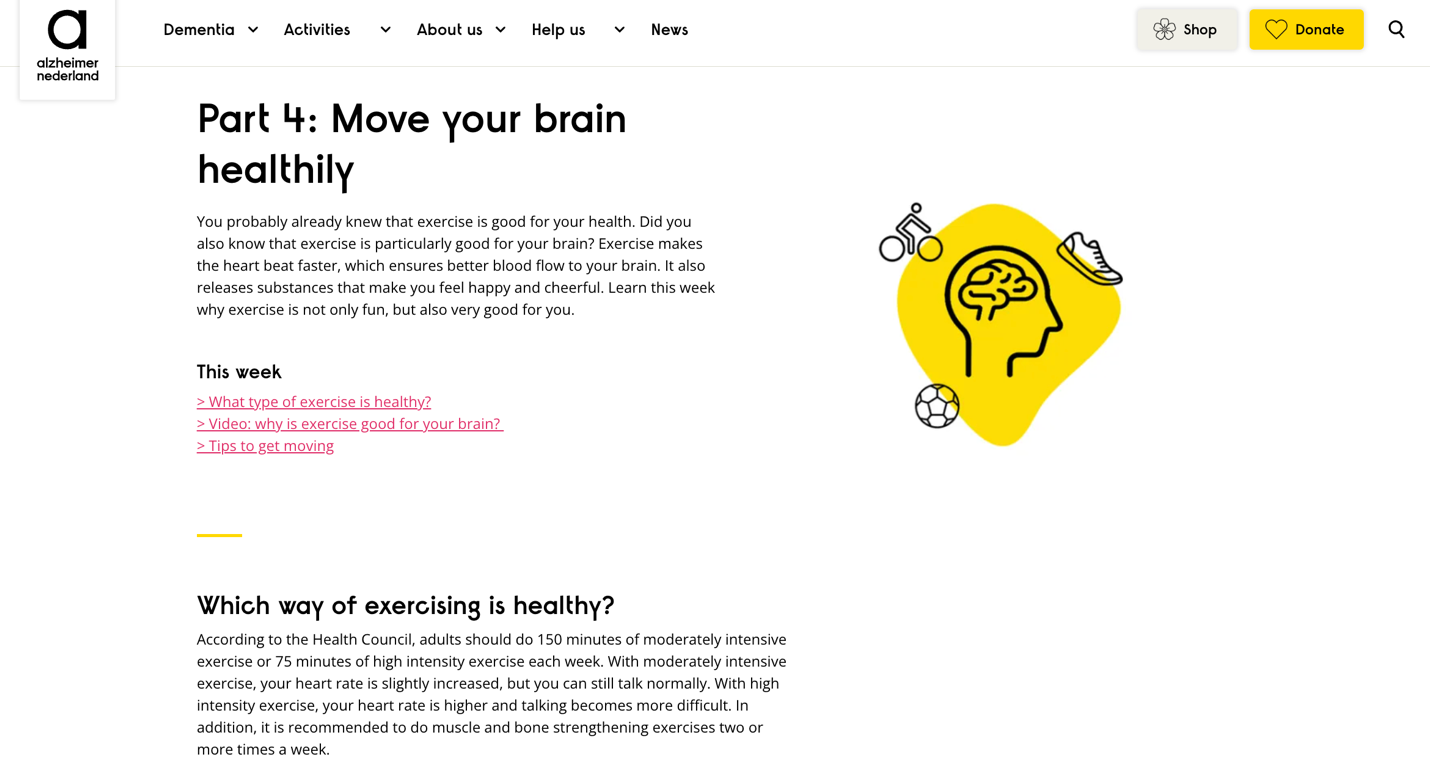
**

**
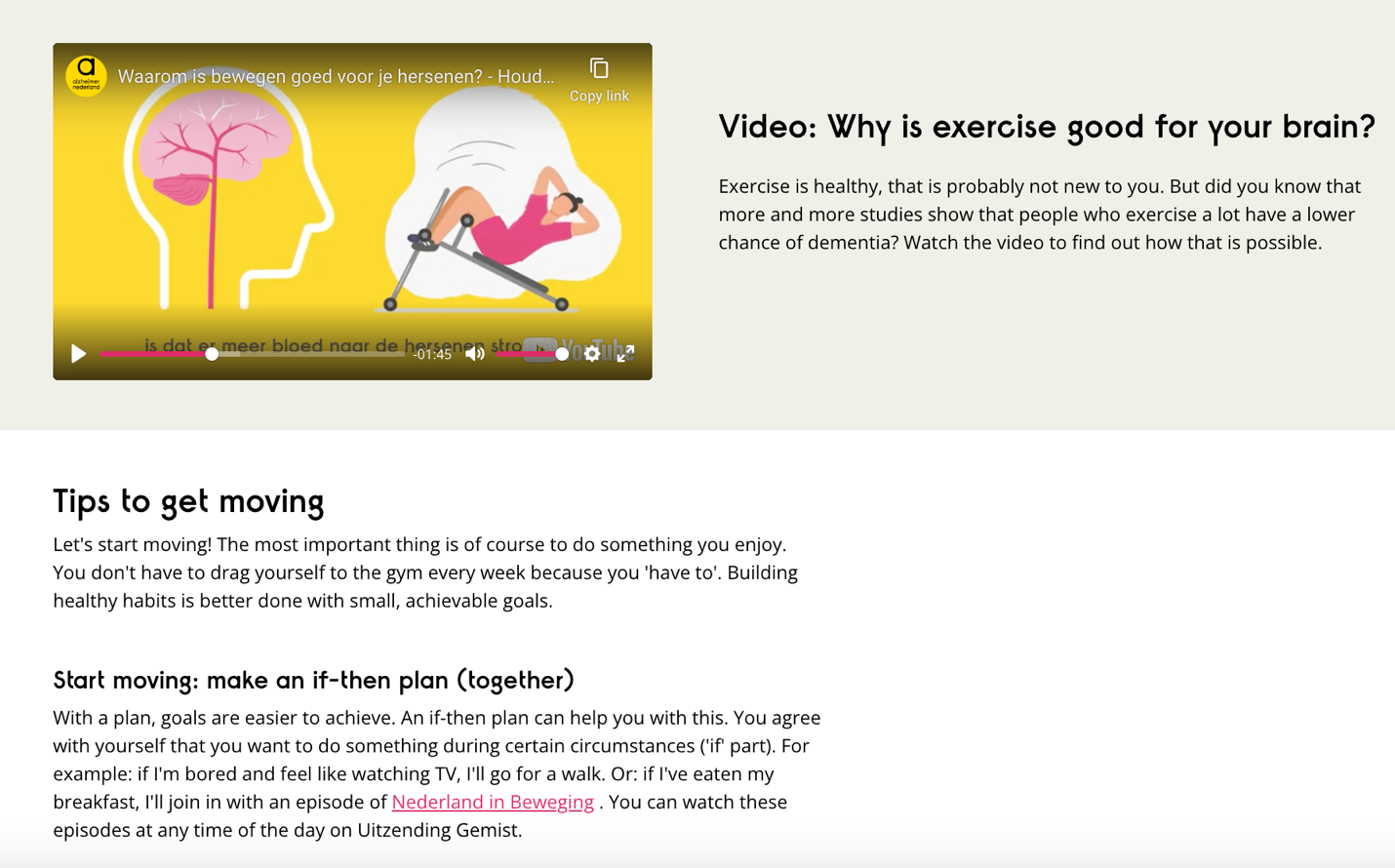
**

Part 5: Your relaxed brain

**
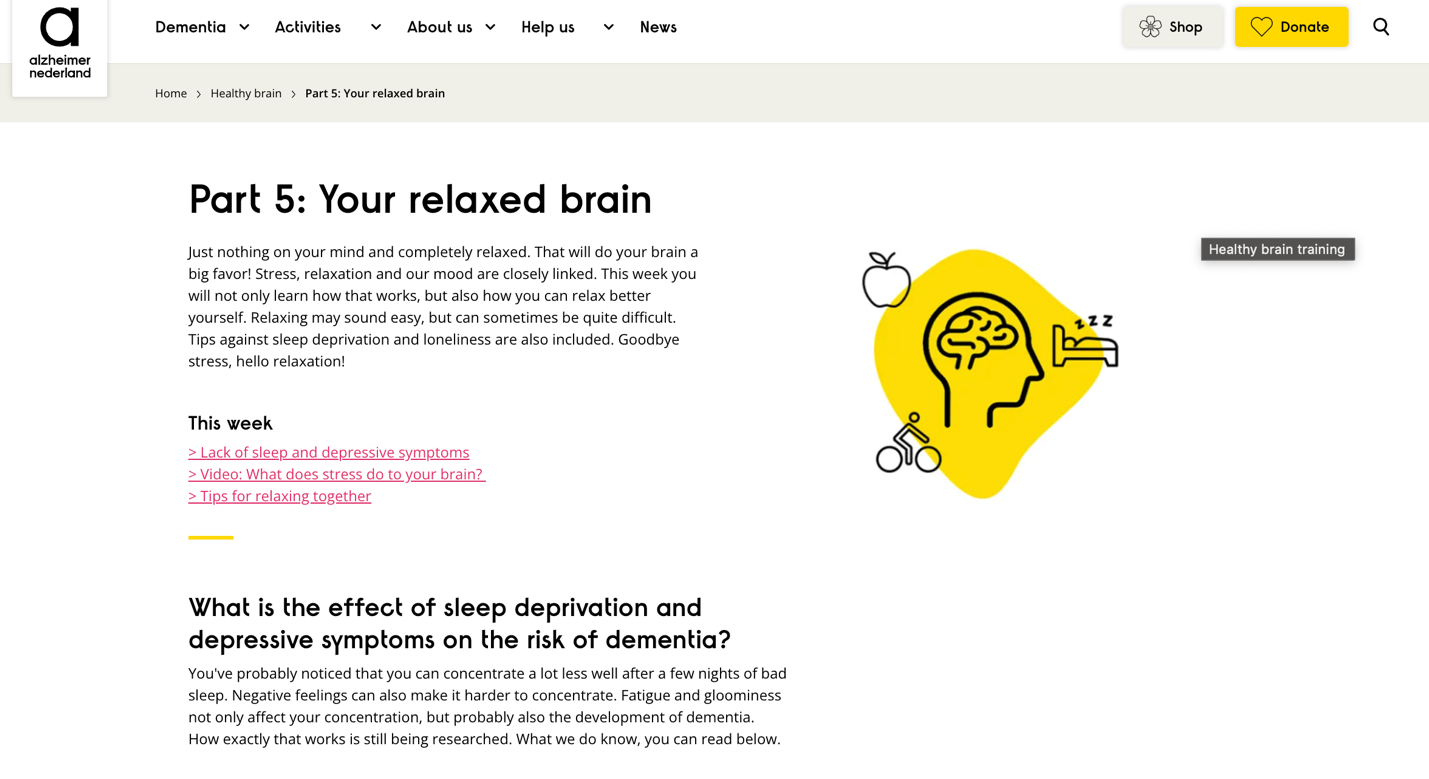
**

**
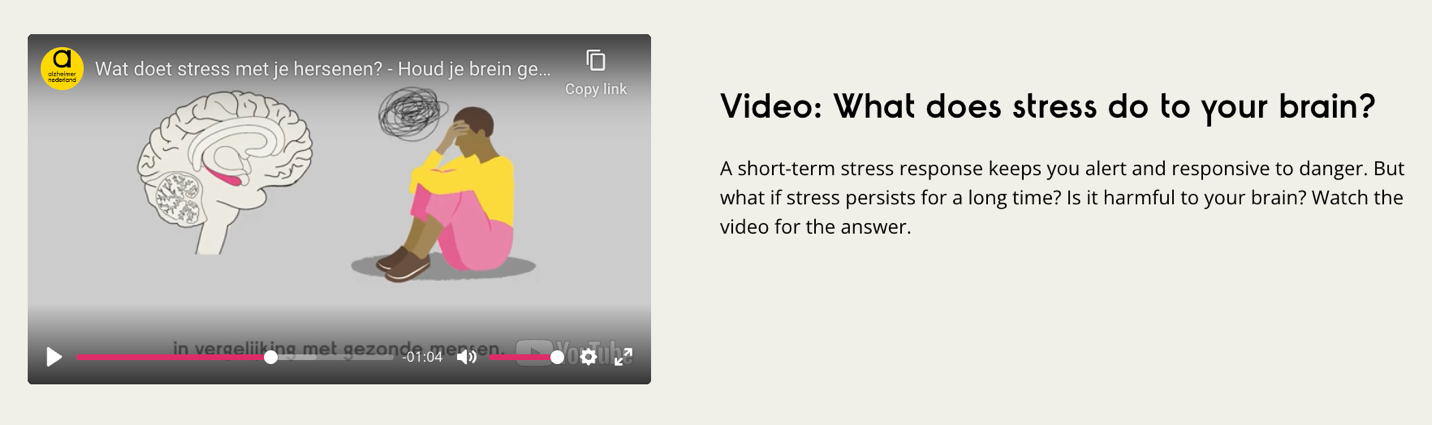
**

Part 6: Good for your heart and brain

**
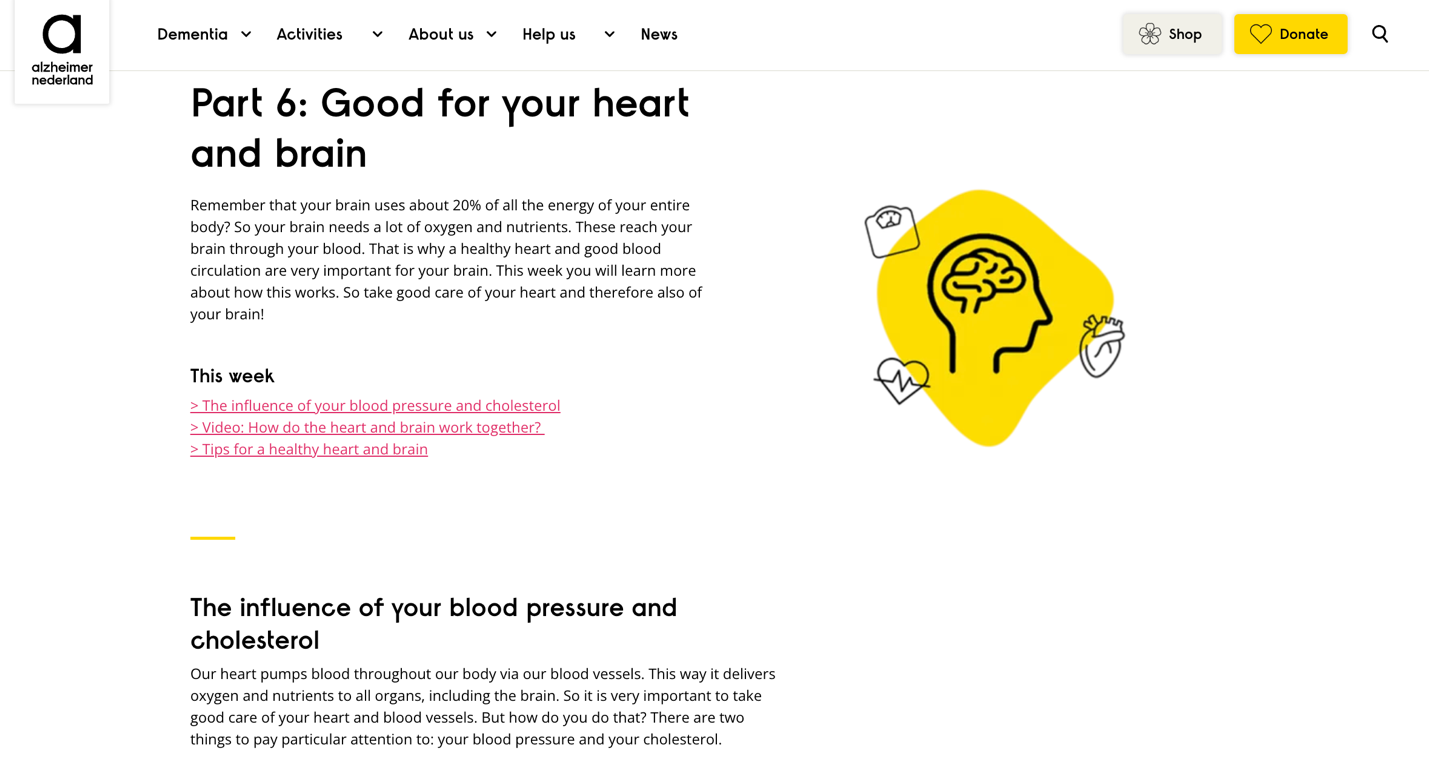
**

**
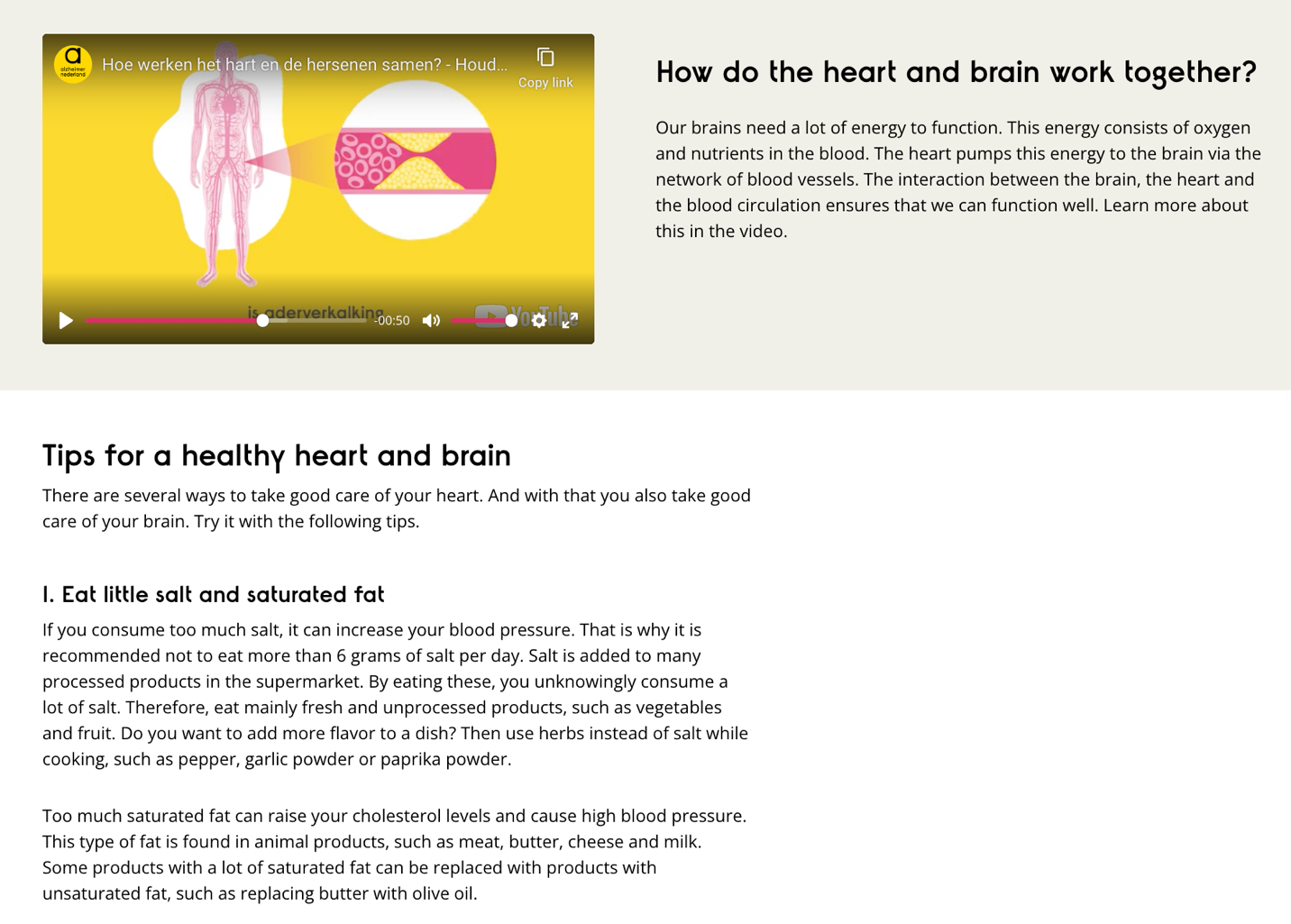
**

Part 7: A fit brain throughout your life

**
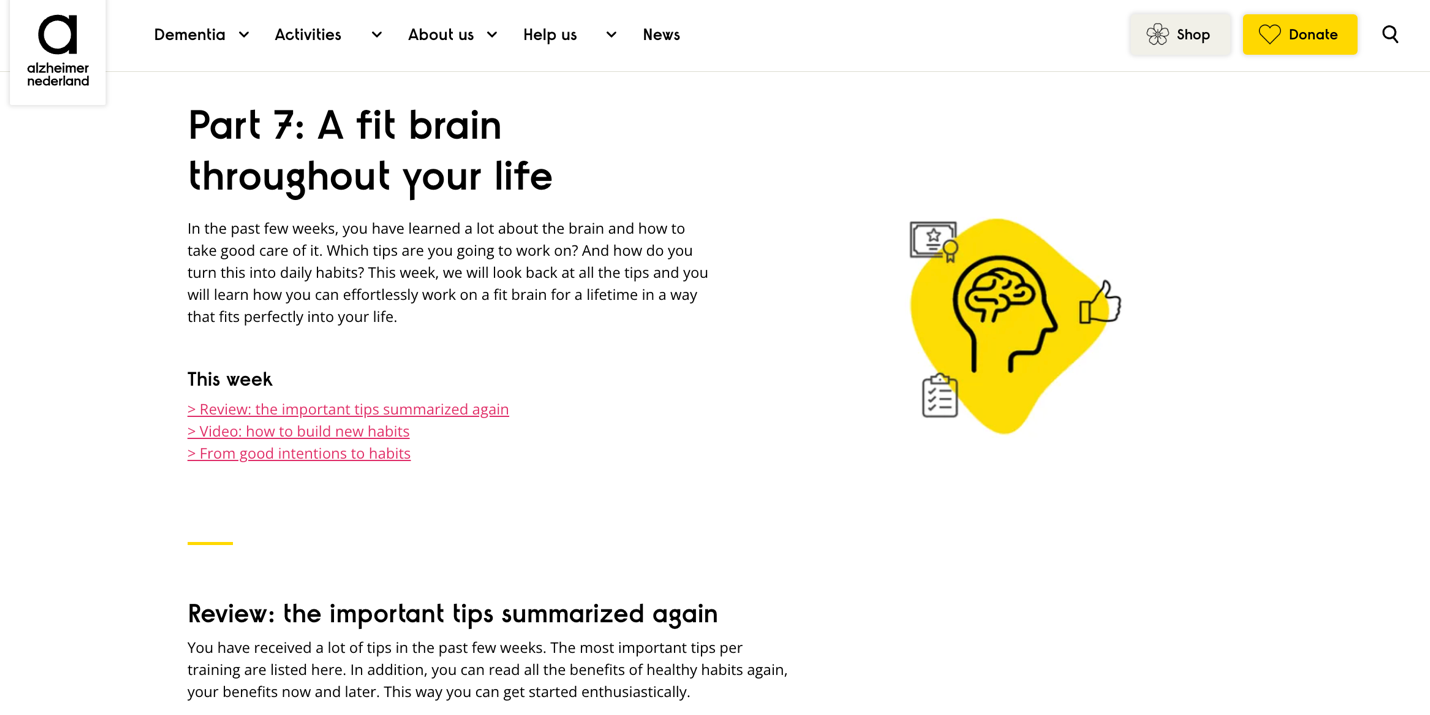
**

**
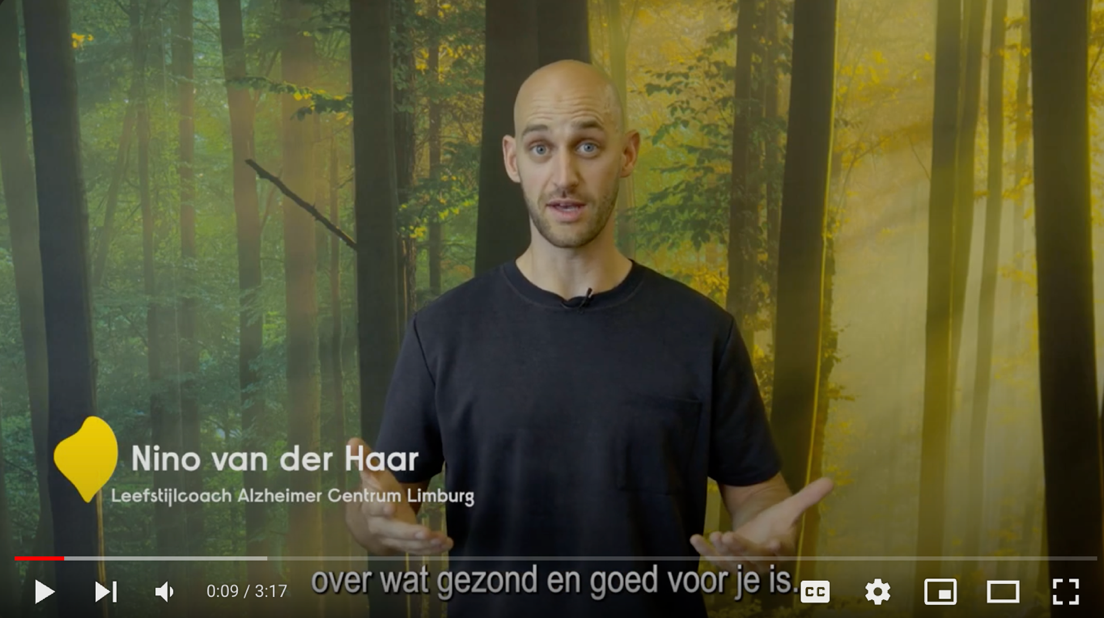
**

Screenshot of the video of part 7, with a lifestyle coach providing tips on how to make sustainable lifestyle changes
