# Supplementary material for: Development and evaluation of a free e-learning program on dementia risk reduction for the general public: A pre-post study
Source: J Alzheimers Dis. 2025 Jan 10;103(4):1075–89. doi: 10.1177/13872877241309112 (PMC12231795; doi:10.1177/13872877241309112)
Supplement: sj-docx-3-alz-10.1177_13872877241309112 - Supplemental material for Development and evaluation of a free e-learning program on dementia risk reduction for the general public: A pre-post study [file sj-docx-3-alz-10.1177_13872877241309112.docx]

**Supplemental Material 3. Overview of data-collection (surveys)**

|  | Baseline | Immediate follow-up | Three-month follow-up |
| --- | --- | --- | --- |
| Demographics and personal familiarity with dementia | x |  |  |
| Knowledge of dementia risk reduction | x | x | x |
| European Prospective Investigation into Cancer and Nutrition (EPIC) physical activity questionnaire items^1, 2^ | x | x | x |
| Cognitive Leisure Activity Scale (CLAS) based items^3^ | x | x | x |
| Questions from the Global Adult Tobacco Survey (GATS)^4^ | x | x | x |
| Questions from the Alcohol Use Disorders Identification Test (AUDIT)^5^ | x | x | x |
| Height & weight | x | Weight only | Weight only |
| Adherence to the Mediterranean diet | x | x | x |
| Social contact satisfaction, sleep quality, and mood | x | x | x |
| Motivation for being physically active | x | x | x |
| Motivation To Stop Scale (MTSS)^6^ | x | x | x |
| Reduce Alcohol Consumption Scale^7^ | x | x | x |
| Motivation for a healthy diet | x | x | x |
| Health conditions (e.g., hypertension, diabetes) | x |  |  |
| Actions taken to better manage health conditions |  | x | x |
| User experience |  | x |  |

Demographics and personal familiarity with dementia

What is your gender?

- Man
- Woman
- Non-binary

What is your age? __

What is the highest degree or level of school you have completed?

- [Six categories according to the Dutch education system]

What is your postal code? __

Have you ever personally known anyone with dementia or have it yourself? You can select multiple answers.

- No, I don't know anyone who has, or had, dementia
- Yes, my job involves / involved working with people who have dementia
- Yes, I have dementia myself
- Yes, my partner, parents (in-law), of children
- Yes, my grandparents of brother/sister
- Yes, a friend(s) I know fairly well
- Yes, a friend(s) or acquaintance(s) I know less well
- Yes, a colleague / someone at my work
- Yes, someone else

Knowledge of dementia risk reduction^8-12^

Please state how much you agree or disagree with the following statements.

*Answer options: Agree strongly*

*Agree*

*Neither agree nor disagree*

*Disagree*

*Disagree strongly*

‘There is nothing anyone can do to reduce their risk of getting dementia’

‘High blood pressure increases your chances of getting dementia’

‘Having a parent with dementia increases the chances of getting dementia’

‘Smoking increases your chances of getting dementia’

‘No or moderate alcohol use lowers your chances of getting dementia’

‘Regular physical activity lowers your chances of getting dementia’

‘Depression increases the chances of getting dementia’

‘The use of painkillers increases your chances of getting dementia’

‘Diabetes increases the chances of getting dementia’

‘Being overweight increases the chances of getting dementia’

‘A mentally active lifestyle lowers the chances of getting dementia’

‘Heart disease increases the chances of getting dementia’

‘Poor personal hygiene increases the chances of getting dementia’

‘Kidney disease increases the chances of getting dementia’

‘High cholesterol increases the chances of getting dementia’

‘Working in a noisy environment increases your chances of getting dementia’

‘A healthy diet lowers the chances of getting dementia’

‘Frequent contact with friends, family, or colleagues lowers the chances of getting dementia’

‘Poor sleep increases the chances of getting dementia’

‘Hearing impairment increases the chances of getting dementia’

***Questions from the Alcohol Use Disorders Identification Test (AUDIT)***^5^

How often do you consume alcoholic beverages?

- Never
- Maximum once per month
- 2-4 times per month
- 2-3 times per week
- 4 or more times per week

*If answer to is not "never":* On the days when you drank alcohol in the past year, how many standard glasses did you typically consume? A standard glass is 25 cl of beer (4-5%), a small glass of wine, or a shot of strong liquor.

- - 1-2 standard glasses
  - 3-4 standard glasses
  - 5-6 standard glasses
  - 7-9 standard glasses
  - 10 or more standard glasses

***Reduce Alcohol Consumption Scale***^7^

*If average alcohol consumption per week is >10 units*:

Which of the following describes you?

- I don't want to cut down on drinking alcohol.
- I think I should cut down on drinking alcohol but don't really want to.
- I want to cut down on drinking alcohol but haven't thought about when.
- I really want to cut down on drinking alcohol but I don't know when I will.
- I want to cut down on drinking alcohol and hope to soon.
- I really want to cut down on drinking alcohol and intend to in the next 3 months.
- I really want to cut down on drinking alcohol and intend to in the next month.

***Questions from the Global Adult Tobacco Survey (GATS)***^4^

Do you currently smoke tobacco?

- Daily
- Less than daily
- Never

*If selected “Daily”:* On average, how many of the following products do you currently smoke each day?

__Cigarettes per day

__Pipes full of tobacco per day

__ Cigars or cigarillos per day

__ Water pipe sessions per day

*If selected “Less than daily”:* On average, how many of the following products do you currently smoke each week?

__ Cigarettes per week

__ Pipes full of tobacco per week

__ Cigars or cigarillos per week

__ Water pipe sessions per week

***Motivation To Stop Scale (MTSS)***^6^

*If currently smokes:* Which of the following describes you?

- I don’t want to stop smoking
- I think I should stop smoking but don’t really want to
- I want to stop smoking but haven’t thought about when
- I really want to stop smoking but I don’t know when I will
- I want to stop smoking and hope to soon
- I really want to stop smoking and intend to in the next 3 months
- I really want to stop smoking and intend to in the next month

Height and weight

What is your height and how much do you weigh?

Height in centimeters __

Weight in kg __

Mediterranean diet

Someone with a Mediterranean diet eats a lot of vegetables, regularly eats fish, pulses (e.g., chickpeas, lentils, beans,…), (olive) oil, wholegrain products (bread, pasta,…), sometimes drinks red wine and eats little sweets, soda, and ready-made meals. To what extent do you recognize your own eating pattern in this?

[Visual analogue scale (VAS) 0-10]

Motivation for a healthy diet

Which of the following describes you?

- I don’t want to eat healthier
- I think I should eat healthier but don’t really want to
- I want to eat healthier but haven’t thought about when
- I want to eat healthier and hope to soon
- I really want to eat healthier and intend to in the next month

***Cognitive Leisure Activity Scale (CLAS) based items***^3^

How often do you participate in each activity? Choose the answer that fits you best.

*Answer options: Never*

*Several times per year*

*Several times per month*

*Once per week*

*Several times per week*

*Daily*

Playing cards, board games, crossword puzzles, jigsaw puzzles, or sudoku’s __

Socializing with friends, family, or colleagues __

Attending a club or group activity or religious service __

Volunteering __

Singing, playing an instrument, painting, drawing or other arts/crafts __

Watching TV or listening to music __

Reading a newspaper, book or magazine __

Going to the theatre, a concert, a museum, or an exhibition __

Attending a conference, lecture, or course __

***European Prospective Investigation into Cancer and Nutrition (EPIC) physical activity questionnaire items***^1, 2^

*Only asked at baseline:* Which description fits your present occupation best?

- Sedentary occupation: You spend most of your time sitting (such as in an office)
- Standing occupation: You spend most of your time standing and walking. However, your work does not require intense physical effort (e.g. shop assistant, hairdresser, guard, etc.)
- Manual work: This involves some physical effort including handling of heavy objects and use of tools (e.g. plumber, electrician, carpenter, etc.)
- Heavy manual work: This implies very vigorous physical activity including handling of very heavy objects (e.g. docker, miner, bricklayer, construction worker, etc.)
- Not applicable

Think about a typical week in the past year[*baseline*]/past month[*follow-up*]. On which of the following activities did you spent time? You can select multiple answers.

- Walking, including walking to work, shopping and leisure time
- Cycling, including cycling to work, shopping and leisure time
- Physical exercise such as fitness, aerobics, swimming, jogging, tennis, etc.

*If selected ‘Walking’*: How many hours did you spend per week on walking, including walking to work, shopping and leisure time?

__ hours in Summer

__ hours in Winter

*If selected ‘Cycling’:* How many hours did you spend per week on cycling, including cycling to work, shopping and leisure time?

__ hours in Summer

__ hours in Winter

*If selected ‘Physical exercise’*: How many hours did you spend per week on physical exercise such as swimming, jogging, tennis, aerobics, etc.?

__ hours in Summer

__ hours in Winter

Motivation for being physically active

Which of the following describes you?

- I don’t want to be more physically active
- I think I should be more physically active but don’t really want to
- I want to be more physically active but haven’t thought about when
- I want to be more physically active and hope to soon
- I really want to be more physically active and intend to in the next month

Social contact satisfaction, sleep quality, and mood

How satisfied are you with your social contacts (family, friends, partner, colleagues, clubs,…)?

[VAS from 0: very unsatisfied, to 10: very satisfied]

How well do you sleep?

[VAS from 0: very poorly, to 10: very good]

How much do you agree with the following statement?

*Generally, I feel cheerful and find life worthwhile.*

[VAS from 0: completely disagree to 10: completely agree]

Health conditions

*Answer options: Yes*

*No*

*I don’t know*

Has your doctor ever told you that your cholesterol is too high?

Has your doctor ever told you that you have a heart or vascular condition (e.g., heart attack, angina, heart failure, stroke, and TIAs)?

Has your doctor ever told you that you have high blood pressure?

Has your doctor ever told you that you have diabetes?

Has your doctor ever told you that you have a chronic kidney disease?

User experience

Which e-training themes did you do? You can select multiple answers.

- Week 1: Your fit brain, about how the brain works and changes in the brain during aging
- Week 2: Challenge your brain, about the importance of mental stimulation
- Week 3: Eat your brain healthy
- Week 4: Move your brain healthy
- Week 5: Your relaxed brain, about the importance of mental wellbeing
- Week 6: Good for heart and brain
- Week 7: Your whole life long a fit brain, about building new healthy habits
- No theme

*For every selected theme:* What did you think about this theme? Please give a score out of 10 points. For example, give a score of 2 out of 10 if you thought the theme was really bad. Or a score of 9 out 10 if you thought the theme was fantastic.

[VAS from 0 to 10]

How would you rate the e-learning? Please rate every the e-learning on every one of the aspects below.

If, for example, you found the training excellent, you can move the slider all the way to the right under "Excellent." If you found the training mediocre, you can place the slider more towards the middle.

[VAS from inferior to excellent]

[VAS from confusing to clear]

[VAS from unpleasant to pleasant]

[VAS from complicated to easy]

[VAS from demotivating to motivating]

[VAS from impractical to practical]

[VAS from ‘does not meet expectations’ to ‘meets expectations’]

[VAS from annoying to enjoyable]

[VAS from boring to exciting]

[VAS from unnattractive to attractive]

[VAS from useless to educational]

Actions taken to better manage health conditions

*Answer options: Yes*

*No*

*No, but I’m planning to*

*No, because this is already well-managed*

*With history of hypertension:* Did you do anything to lower your blood pressure?

*With history of elevated cholesterol:* Did you do anything to lower your cholesterol?

*With history of diabetes:* Did you do anything to better manage your glyceamia?

**References**

1. Cust AE, Smith BJ, Chau J, et al. Validity and repeatability of the EPIC physical activity questionnaire: a validation study using accelerometers as an objective measure. *Int J Behav Nutr Phys Act* 2008; 5: 33.

2. Haftenberger M, Schuit AJ, Tormo MJ, et al. Physical activity of subjects aged 50-64 years involved in the European Prospective Investigation into Cancer and Nutrition (EPIC). *Public Health Nutr* 2002; 5: 1163-1176.

3. Galvin JE, Tolea MI and Chrisphonte S. The Cognitive & Leisure Activity Scale (CLAS): A new measure to quantify cognitive activities in older adults with and without cognitive impairment. *Alzheimers Dement (N Y)* 2021; 7: e12134.

4. Global Adult Tobacco Survey Collaborative Group. *Tobacco Questions for Surveys: A Subset of Key Questions from the Global Adult Tobacco Survey (GATS)*. Atlanta, GA, 2011.

5. Saunders JB, Aasland OG, Babor TF, et al. Development of the Alcohol Use Disorders Identification Test (AUDIT): WHO Collaborative Project on Early Detection of Persons with Harmful Alcohol Consumption--II. *Addiction* 1993; 88: 791-804.

6. Kotz D, Brown J and West R. Predictive validity of the Motivation To Stop Scale (MTSS): A single-item measure of motivation to stop smoking. *Drug Alcohol Depend* 2013; 128: 15-19.

7. de Vocht F, Brown J, Beard E, et al. Motivation to reduce alcohol consumption and subsequent attempts at reduction and changes in consumption in increasing and higher-risk drinkers in England: a prospective population survey. *Addiction* 2018; 113: 817-827.

8. Heger I, Kohler S, van Boxtel M, et al. Raising awareness for dementia risk reduction through a public health campaign: a pre-post study. *BMJ Open* 2020; 10: e041211.

9. Van Asbroeck S, van Boxtel MPJ, Steyaert J, et al. Increasing knowledge on dementia risk reduction in the general population: Results of a public awareness campaign. *Prev Med* 2021; 147: 106522.

10. Kjelvik G, Rokstad AMM, Stuebs J, et al. Public knowledge about dementia risk reduction in Norway. *BMC Public Health* 2022; 22: 2046.

11. Zulke AE, Luppa M, Kohler S, et al. Knowledge of risk and protective factors for dementia in older German adults A population-based survey on risk and protective factors for dementia and internet-based brain health interventions. *PLoS One* 2022; 17: e0277037.

12. Rosenau C, Kohler S, Soons LM, et al. Umbrella review and Delphi study on modifiable factors for dementia risk reduction. *Alzheimers Dement* 2024; 20: 2223-2239.
