# Supplementary material for: Development and evaluation of a free e-learning program on dementia risk reduction for the general public: A pre-post study
Source: J Alzheimers Dis. 2025 Jan 10;103(4):1075–89. doi: 10.1177/13872877241309112 (PMC12231795; doi:10.1177/13872877241309112)
Supplement: sj-docx-4-alz-10.1177_13872877241309112 - Supplemental material for Development and evaluation of a free e-learning program on dementia risk reduction for the general public: A pre-post study [file sj-docx-4-alz-10.1177_13872877241309112.docx]

**Supplemental Material 4: Detailed results on motivation for health behaviors**

At baseline in the entire sample, motivation for a healthy diet was higher among younger individuals (b_age_=-0.05, p<0.001) and lower among individuals who already adhered more to the Mediterranean diet (b=-0.30, p<0.001). There was no change over time in motivation for a healthy diet (b_immediate_=0.30, p=0.058 and b_3 months_=-0.13, p=0.467). When limiting the analysis to individuals with poor adherence to the Mediterranean diet (self-rating<6), motivation for a healthy diet at baseline was, again, higher among younger individuals (b_age_=-0.06, p=0.013), but conversely, was higher among individuals who already adhered more to the Mediterranean diet (b=0.42, p=0.045). Motivation for a healthy diet did not change over time among those with poor adherence to the Mediterranean diet at baseline (b_immediate_=0.60, p=0.070 and b_3 months_=-0.001, p=0.998).

Further, in the entire sample, motivation for physical activity at baseline was higher among younger individuals (b_age_=-0.03, p=0.004), and among individuals who were physically inactive at baseline (b=1.00, p<0.001). Moreover, motivation was higher by immediate follow-up (b=3.14, p=0.003) but not after three months. However, the increase in motivation for physical activity at immediate follow-up was larger among younger compared to older individuals (b_age × time_ =-0.04, p=0.005), and among low- or medium educated individuals compared to highly educated individuals (b=-0.67, p=0.043). This age × time interaction was significant over all time points combined (p=0.009). When limiting the analysis to individuals who were physically inactive at baseline, motivation for physical activity was again higher for younger individuals (b_age_=-0.04, p=0.024). An increase in motivation by immediate follow-up was also observed (b=6.28, p<0.001) and was no longer apparent after three months. Moreover, the increase in motivation by immediate follow-up was smaller in highly educated individuals (b_education × time_=-1.86, p=0.001), and in older individuals (b_age × time_=-0.07, p=0.003). Over all time points combined, both age × time and education × time interactions were significant (p=0.005, and p=0.005 respectively).

For individuals who drank too much alcohol (>7 standard units/week) or individuals who smoked, their motivation for reducing alcohol consumption or quitting smoking, also did not change over time. Notably, sample sizes were also small (n_excessive drinking_=32, n_smoked_=10 at immediate follow-up).
