# Supplementary material for: Development and evaluation of a free e-learning program on dementia risk reduction for the general public: A pre-post study
Source: J Alzheimers Dis. 2025 Jan 10;103(4):1075–89. doi: 10.1177/13872877241309112 (PMC12231795; doi:10.1177/13872877241309112)
Supplement: sj-docx-5-alz-10.1177_13872877241309112 - Supplemental material for Development and evaluation of a free e-learning program on dementia risk reduction for the general public: A pre-post study [file sj-docx-5-alz-10.1177_13872877241309112.docx]

**Supplemental Material 5. E-learning usage data**

**Supplemental Table 1. E-learning e-mail engagement stratified by research participation status**

|  | | Participants, n (%) | Non-participating users, n (%) |
| --- | --- | --- | --- |
| E-mail 1  How the brain works | Sent e-mails | 477 (NA) | 1175 (NA) |
|  | Number of opens | 458 (96.0) | 982 (83.6) |
|  | Clicks to quiz | 236 (49.5) | 681 (58.0) |
|  | Clicks to webpage | 22 (4.6) | 81 (6.9) |
|  | Clicks to question of the week | 22 (4.6) | 177 (15.1) |
| E-mail 2  Cognitive activity | Sent e-mails | 477 (NA) | 810 (NA) |
|  | Number of opens | 471 (98.7) | 608 (75.1) |
|  | Clicks to quiz | 275 (57.7) | 379 (46.8) |
|  | Clicks to webpage | 21 (4.4) | 47 (5.8) |
|  | Clicks to question of the week | 34 (7.1) | 57 (7.0) |
| E-mail 3  Healthy diet | Sent e-mails | 477 (NA) | 795 (NA) |
|  | Number of opens | 432 (90.6) | 615 (77.4) |
|  | Clicks to quiz | 340 (71.3) | 358 (45.0) |
|  | Clicks to webpage | 23 (4.8) | 50 (6.3) |
|  | Clicks to question of the week | 49 (10.3) | 79 (9.9) |
| E-mail 4  Physical activity | Sent e-mails | 477 (NA) | 787 (NA) |
|  | Opened e-mail | 427 (89.5) | 566 (71.9) |
|  | Clicks to quiz | 316 (66.2) | 300 (38.1) |
|  | Clicks to webpage | 29 (6.1) | 30 (3.8) |
|  | Clicks to question of the week | 30 (6.3) | 25 (3.2) |
| E-mail 5 Mental wellbeing | Sent e-mails | 477 (NA) | 782 (NA) |
|  | Opened e-mail | 414 (86.8) | 553 (70.7) |
|  | Clicks to quiz | 292 (61.2) | 281 (35.9) |
|  | Clicks to webpage | 40 (8.4) | 50 (6.4) |
|  | Clicks to question of the week | 22 (4.6) | 25 (3.2) |
| E-mail 6  Good for heart and brain | Sent e-mails | 477 (NA) | 775 (NA) |
|  | Opened e-mail | 405 (84.9) | 541 (69.8) |
|  | Clicks to quiz | 293 (61.4) | 260 (33.5) |
|  | Clicks to webpage | 26 (5.5) | 30 (3.9) |
|  | Clicks to question of the week | 25 (5.2) | 32 (4.1) |
| E-mail 7 Sustainable behavior change | Sent e-mails | 477 (NA) | 770 (NA) |
|  | Opened e-mail | 401 (84.1) | 533 (69.2) |
|  | Clicks to quiz | 289 (60.6) | 225 (29.2) |
|  | Clicks to webpage | 22 (4.6) | 14 (1.8) |
|  | Clicks to question of the week | 41 (8.6) | 45 (5.8) |

**Supplemental Figure 1. E-learning e-mail engagement stratified by research participation status**

**Supplemental Table 2. Quiz engagement in participants and non-participating users**

|  | | Views, n | Starts, n (%) | Completions, n (%) | Time spent (s) |
| --- | --- | --- | --- | --- | --- |
| Theme 1 | Participants | 449 | 192 (42.8) | 171 (38.1) | 140 |
|  | Non-participating users | 34000 | 17500 (51.5) | 14800 (43.5) | 133 |
| Theme 2 | Participants | 640 | 279 (43.6) | 246 (38.4) | 168 |
|  | Non-participating users | 27500 | 13900 (50.5) | 11000 (40.0) | 146 |
| Theme 3 | Participants | 714 | 303 (42.4) | 280 (39.2) | 167 |
|  | Non-participating users | 23300 | 11900 (51.1) | 9940 (42.7) | 150 |
| Theme 4 | Participants | 484 | 218 (45.0) | 207 (42.8) | 147 |
|  | Non-participating users | 18200 | 9290 (51.0) | 7780 (42.7) | 135 |
| Theme 5 | Participants | 659 | 307 (46.6) | 262 (39.8) | 136 |
|  | Non-participating users | 17800 | 9080 (51.0) | 7250 (40.7) | 144 |
| Theme 6 | Participants | 623 | 252 (40.4) | 237 (38.0) | 131 |
|  | Non-participating users | 16100 | 8050 (50.0) | 6900 (42.9) | 129 |
| Theme 7 | Participants | 598 | 236 (39.5) | 219 (36.6) | 122 |
|  | Non-participating users | 13300 | 6650 (50.0) | 5780 (43.5) | 115 |

Non-participating users includes everyone since implementation of quiz, not just non-participating users during the time period of the study. Start and completion proportions were calculated relative to total views.

**Supplemental Table 3. Engagement with the e-learning quiz and webpage among *non-participating users* during study period**

|  |  | Total users | Sessions | Average session time (s) | Engagement percentage (%) |
| --- | --- | --- | --- | --- | --- |
| Theme 1: How the brain works | Quiz | 8429 | 10025 | 192 | 86.9 |
|  | Webpage | 3758 | 4791 | 394 | 83.4 |
|  | Video | 1487 | NA | NA | NA |
| Theme 2: Cognitive activity | Quiz | 6772 | 8065 | 141 | 86.2 |
|  | Webpage | 3278 | 3995 | 321 | 86.7 |
|  | Video | 1051 | NA | NA | NA |
| Theme 3: Healthy diet | Quiz | 6049 | 7114 | 119 | 84.0 |
|  | Webpage | 1832 | 2140 | 297 | 86.6 |
|  | Video | 529 | NA | NA | NA |
| Theme 4: Physical activity | Quiz | 5099 | 5828 | 149 | 81.8 |
|  | Webpage | 1523 | 1867 | 444 | 83.0 |
|  | Video | 453 | NA | NA | NA |
| Theme 5: Mental wellbeing | Quiz | 4387 | 5016 | 125 | 82.2 |
|  | Webpage | 1149 | 1344 | 286 | 88.8 |
|  | Video | 348 | NA | NA | NA |
| Theme 6: Good for heart and brain | Quiz | 3964 | 4571 | 102 | 81.6 |
|  | Webpage | 1065 | 1248 | 268 | 86.1 |
|  | Video | 252 | NA | NA | NA |
| Theme 7: Sustainable behavior change | Quiz | 2609 | 2902 | 98 | 85.4 |
|  | Webpage | 1021 | 1169 | 167 | 89.2 |
|  | Video | 108 | NA | NA | NA |
